# Effect of Financial Incentives for Process, Outcomes, or Both on Cholesterol Level Change: A Randomized Clinical Trial

**DOI:** 10.1001/jamanetworkopen.2021.21908

**Published:** 2021-10-04

**Authors:** Peter P. Reese, Iwan Barankay, Mary Putt, Louise B. Russell, Jiali Yan, Jingsan Zhu, Qian Huang, George Loewenstein, Rolf Andersen, Heidi Testa, Adam S. Mussell, David Pagnotti, Lisa E. Wesby, Karen Hoffer, Kevin G. Volpp

**Affiliations:** 1 Department of Biostatistics, Epidemiology and Informatics, Perelman School of Medicine, University of Pennsylvania, Philadelphia; 2 Department of Medicine, Perelman School of Medicine, University of Pennsylvania, Philadelphia; 3 Leonard Davis Institute, University of Pennsylvania, Philadelphia; 4 Center for Health Incentives and Behavioral Economics, University of Pennsylvania, Philadelphia; 5 Department of Medical Ethics and Health Policy, Perelman School of Medicine, University of Pennsylvania, Philadelphia; 6 Department of Management, Department of Business Economics and Public Policy, The Wharton School, University of Pennsylvania, Philadelphia; 7 Department of Social and Decision Sciences, Carnegie Mellon University, Pittsburgh, Pennsylvania; 8 The Heart Group, Lancaster General Health/Penn Medicine, Lancaster, Pennsylvania; 9 Research Institute, Lancaster General Health/Penn Medicine, Lancaster, Pennsylvania

## Abstract

**Importance:**

Financial incentives may improve health behaviors. It is unknown whether incentives are more effective if they target a key process (eg, medication adherence), an outcome (eg, low-density lipoprotein cholesterol [LDL-C] levels), or both.

**Objective:**

To determine whether financial incentives awarded daily for process (adherence to statins), awarded quarterly for outcomes (personalized LDL-C level targets), or awarded for process plus outcomes induce reductions in LDL-C levels compared with control.

**Design, Setting, and Participants:**

A randomized clinical trial was conducted from February 12, 2015, to October 3, 2018; data analysis was performed from October 4, 2018, to May 27, 2021, at the University of Pennsylvania Health System, Philadelphia. Participants included 764 adults with an active statin prescription, elevated risk of atherosclerotic cardiovascular disease, suboptimal LDL-C level, and evidence of imperfect adherence to statin medication.

**Interventions:**

Interventions lasted 12 months. All participants received a smart pill bottle to measure adherence and underwent LDL-C measurement every 3 months. In the process group, daily financial incentives were awarded for statin adherence. In the outcomes group, participants received incentives for achieving or sustaining at least a quarterly 10-mg/dL LDL-C level reduction. The process plus outcomes group participants were eligible for incentives split between statin adherence and quarterly LDL-C level targets.

**Main Outcomes and Measures:**

Change in LDL-C level from baseline to 12 months, determined using intention-to-treat analysis.

**Results:**

Of the 764 participants, 390 were women (51.2%); mean (SD) age was 62.4 (10.0) years, 310 (40.6%) had diabetes, 298 (39.0%) had hypertension, and mean (SD) baseline LDL-C level was 138.8 (37.6) mg/dL. Mean LDL-C level reductions from baseline to 12 months were −36.9 mg/dL (95% CI, −42.0 to −31.9 mg/dL) among control participants, −40.0 mg/dL (95% CI, −44.7 to −35.4 mg/dL) among process participants, −41.6 mg/dL (95% CI, −46.3 to −37.0 mg/dL) among outcomes participants, and −42.8 mg/dL (95% CI, −47.4 to −38.1 mg/dL) among process plus outcomes participants. In exploratory analysis among participants with diabetes and hypertension, no spillover effects of incentives were detected compared with the control group on hemoglobin A_1c_ level and blood pressure over 12 months.

**Conclusions and Relevance:**

In this randomized clinical trial, process-, outcomes-, or process plus outcomes–based financial incentives did not improve LDL-C levels vs control.

**Trial Registration:**

ClinicalTrials.gov Identifier: NCT02246959

## Key Points

**Question:**

Do financial incentives rewarding process (statin adherence), outcomes (reduction in low-density lipoprotein cholesterol), or both reduce low-density lipoprotein cholesterol levels compared with control?

**Findings:**

In this randomized clinical trial, 764 participants with an active statin prescription, elevated cardiovascular disease risk, suboptimal cholesterol levels, and imperfect statin adherence received financial incentives for 12 months. Reduction in low-density lipoprotein cholesterol levels from baseline to 12 months, the primary outcome, was similar between the intervention groups vs the control group.

**Meaning:**

The findings of this trial indicate that, compared with control, process incentives (statin adherence), outcome incentives (low-density lipoprotein cholesterol level goals), or a combination of both may not improve low-density lipoprotein cholesterol levels.

## Teaser

This randomized clinical trial evaluates the use of financial incentives to improve adherence to statin therapy and achieve a decrease in low-density lipoprotein cholesterol levels in individuals at risk for atherosclerotic cardiovascular disease.

## Introduction

Atherosclerotic cardiovascular disease (ASCVD) remains the number 1 cause of death globally.^1^ Use of statins (3-hydroxy-3-methylglutaryl coenzyme A reductase inhibitors) has improved cholesterol levels and lowered ASCVD mortality in clinical trials.^2,3^ Statins are inexpensive and generally have manageable adverse effects.^4,5^ Nonadherence is common, however, even among patients who previously experienced a myocardial infarction, and contributes to worse health outcomes.^6,7^ Statin nonadherence is therefore a serious barrier to realizing public health gains in the treatment of patients at risk for ASCVD-associated events.

Financial incentives have been shown to improve some health behaviors, such as physical activity and smoking cessation, by providing an immediate reward for an action that would otherwise offer only the possibility of future health benefits.^8,9,10,11,12,13,14,15,16^ In studies of other health behaviors, such as medication adherence and weight loss, however, financial incentives have not consistently improved outcomes.^17,18,19,20,21,22^ The effectiveness of financial incentives may depend on the design, timing of feedback, and the nature of the behavior being incented, as well as the ability to measure the targeted health behavior accurately and nonintrusively.^23,24^ Statins are taken daily, which requires organization, and do not perceptibly improve short-term quality of life, so daily financial incentives for taking the statin (a process-of-care incentive) could help nonadherent users overcome present bias (the cognitive tendency to value behaviors with immediate rewards while undervaluing longer-term consequences).^25^ For statin users who face complex social conditions and multiple comorbidities, incentives might help them stay focused on the longer-term health benefits of reducing cholesterol levels. Recent advances in smart pill bottle technology have facilitated the measurement of medication adherence,^26^ but their use may be difficult to integrate into a patient’s routines.^27^ In addition, incentives focused only on statin adherence might cause patients to neglect other important health behaviors, such as diet or exercise, or signal to patients that statins should take priority over other important medications,^28^ causing unintended spillover effects on nontargeted health conditions.^29^

Given these concerns, an intervention directly rewarding a surrogate health outcome, such as lowering low-density lipoprotein cholesterol (LDL-C) levels (an outcome incentive), could be appealing compared with daily adherence incentives.^30^ Low-density lipoprotein cholesterol lies on the causal pathway between statin use and clinical outcomes, such as a myocardial infarction or death. Furthermore, LDL-C levels can be improved by diet, exercise, and weight loss as well as statin therapy, so a cholesterol-targeted intervention could allow patients to choose their preferred healthy behaviors. A disadvantage of incentivizing LDL-C level improvement is that any change in behavior would likely take weeks to affect LDL-C levels and trigger a financial reward. Furthermore, because many health outcomes, such as LDL-C level lowering, are measured infrequently in usual care, the feedback to patients would take place sporadically. Patients who did not qualify for an outcome incentive might feel that they lacked timely guidance or the ability to take beneficial steps to earn incentives.^31^

Direct comparison of process and outcome incentives for medication adherence is novel.^32,33^ We designed a 4-group randomized clinical trial to test the efficacy of 12-month interventions involving financial incentives for process, outcomes, and a combination of both for statin users with evidence of nonadherence. The primary outcome was a change in LDL-C levels over 12 months.

## Methods

### Design and Participants

From February 12, 2015, through October 18, 2018, we conducted a 4-group randomized clinical trial aimed at improving LDL-C levels. We analyzed the data from October 4, 2018, to May 27, 2021. Financial incentives were offered to participants in 3 groups for 12 months. All participants received smart pill bottles that measured openings and were wirelessly integrated with a technology platform (Penn Way to Health).^34^ The protocol (Supplement 1) was approved by the institutional review boards of the University of Pennsylvania and Lancaster General Hospital and the study’s data safety monitoring board. Recruitment and consent were conducted via telephone and we obtained a waiver of written consent.

Participants were recruited from the University of Pennsylvania Health System (Penn Medicine) and cardiology practices at Lancaster General Hospital, a Penn Medicine affiliate.^35^ Penn Medicine recruitment primarily involved patients in the Philadelphia metropolitan area, and Lancaster General serves rural southeastern Pennsylvania. The intended initial sample size was met based on active recruitment at both sites.

The primary outcome was change in LDL-C levels from baseline to 12 months (346-388 days postrandomization). Low-density lipoprotein cholesterol levels were measured at baseline and 3, 6, 9, 12, and 18 months. Prespecified secondary outcomes included change in LDL-C level from baseline to 18 months and measured adherence over the 12-month intervention period (the proportion of 360 days in which the pill bottle registered an opening).

We also conducted a prespecified analysis to explore spillover effects of financial incentives on non–LDL-C outcomes. Negative spillover could reflect increased attention among patients to LDL-C level control, resulting in decreased attention to other health conditions; positive spillover might result from patients increasing their attention to behaviors related to their health in general. To estimate changes in glucose level control and blood pressure among patients with diagnoses of diabetes and/or hypertension, we collected systolic blood pressure and hemoglobin A_1c_ (HbA_1c_) data using the electronic medical record during usual care (eMethods 1 in Supplement 2 provides details).

Throughout the trial, eligibility was limited to adults (age ≥18 years) with a statin prescription and evidence of low statin adherence. Initially, eligibility was limited to individuals with LDL-C levels greater than 130 mg/dL (to convert to millimoles per liter, multiply by 0.0259), plus diabetes and/or ASCVD. Given updates to national guidelines about patients for whom statins should be prescribed after the trial started, we modified eligibility criteria to include adults receiving statin therapy, with low statin adherence, who also had LDL-C levels greater than 190 mg/dL and/or LDL-C levels greater than 100 mg/dL plus a diagnosis of ASCVD, and/or an American College of Cardiology/American Heart Association Task Force 10-year cardiovascular disease risk score of at least 7.5%, and/or diabetes and age between 40 and 75 years.^36^ We excluded individuals younger than 18 years, those with contraindications to statin use or adverse effects due to statins, such as active or progressive liver disease, those receiving PCSK9 inhibitors, those with comorbidities expected to lead to death within a short period (eg, metastatic cancer), and those who did not or could not provide informed consent. We also excluded individuals who self-reported perfect adherence to the statin (eMethods 2 in Supplement 2).

**Figure 1.**
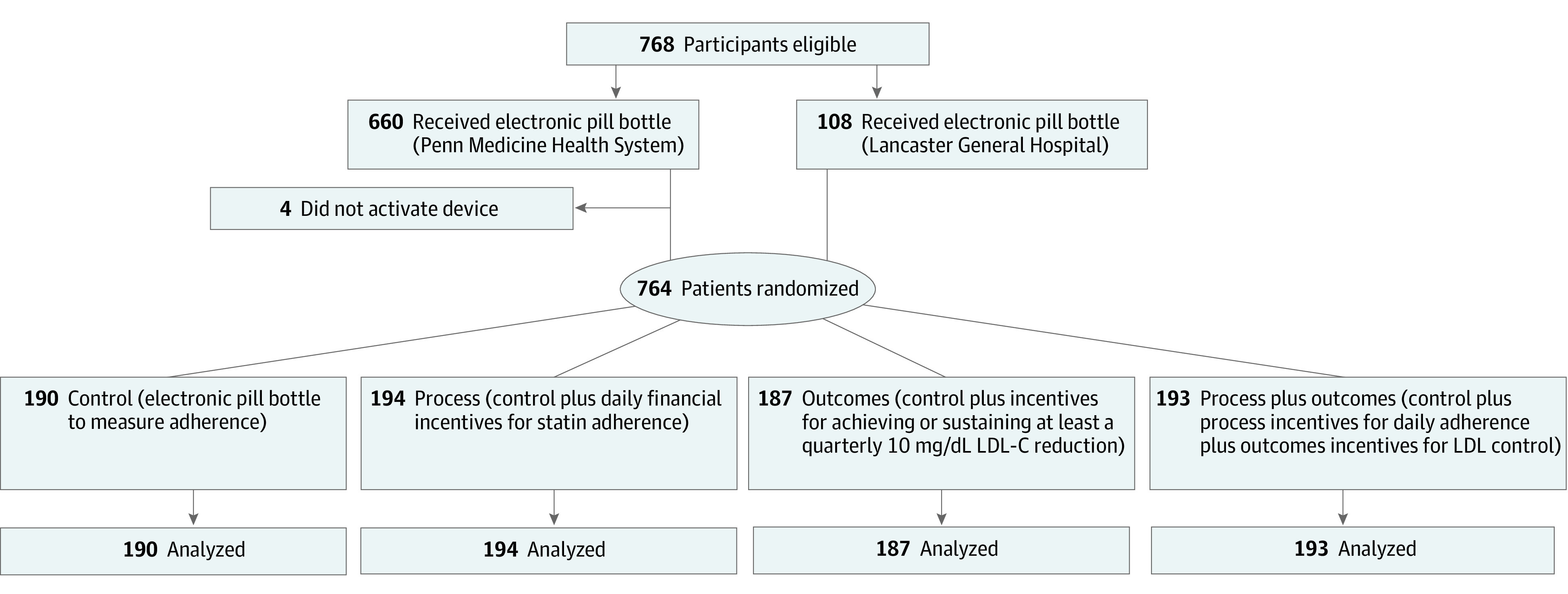
Consolidated Standards of Reporting Trials Diagram LDL-C indicates low-density lipoprotein cholesterol. SI conversion factor: To convert LDL-C to millimoles per liter, multiply by 0.0259.

We invited 7406 individuals to be screened for the trial based on medical record review (eFigure 1 in Supplement 2).^34,35^ Through the Way to Health website, by telephone, or in person, 1719 potential participants completed informed consent and a screening survey to confirm eligibility (Figure 1). Invitees were queried about demographic characteristics, health, readiness for behavioral change, income to meet financial challenges, ability to manipulate fractions and other mathematical functions, willingness to take risks, statin adherence, and other domains (eMethods 3 in Supplement 2).

A total of 768 participants were eligible, consented, and received smart pill bottles. Randomization to study group took place when a pill bottle was activated. A total of 764 individuals activated their bottles and were block randomized via the Way to Health platform using computer-generated sequences, with equal allocation to each group.^34^ Random block sizes were 4 or 8, stratified by site (Penn Medicine or Lancaster General Hospital), sex, self-reported pretax household income (<$50 000, ≥$50 000, or prefer not to answer), and 1 of 3 models of smart pill bottles (eMethods 4 in Supplement 2). Investigators and analytical staff, but not participants, were blinded to assignment.

### Interventions

All participants received up to $325 for completing study milestones (enrollment plus 5 visits for LDL-C level measurement). The control group received no further intervention.

Over a 12-month period, individuals in the 3 intervention groups were eligible for financial incentives based on their daily statin adherence (process group), LDL-C value every 3 months (outcomes group), or both (process plus outcomes group). We delivered statin adherence incentives as a sweepstakes to invoke people’s tendency to overestimate small probabilities.^37,38^ One daily bottle opening made the process and process plus outcomes participants eligible for that day’s incentive. Incentives for LDL-C level reduction in the outcomes and process plus outcomes groups were delivered without a sweepstakes. A fully adherent participant in a financial incentives group could earn, on average, $1.40 per day.

Participants in the outcomes groups received $126 each time their LDL-C level had decreased at least 10 mg/dL in the 3 months since the last visit. During the study year, these payments were equivalent to the expected value of the process group. A participant who achieved an LDL-C level reduction of 20 mg/dL or more during 1 quarter and maintained that reduction the next quarter would receive the outcome incentive at the end of both quarters, even if no further reduction in LDL-C levels took place during the second quarter.

Participants in the process plus outcomes group were eligible for daily sweepstake rewards, but with half the expected monetary value of the process group, and for 3-monthly LDL-C rewards at half the value of the outcomes group. Participants in all 3 intervention groups had the opportunity to earn $504 if they met all adherence and/or LDL-C level reduction targets for their assigned group (eMethods 5 in Supplement 2 provides details about implementation of financial incentives).

### Statistical Analysis

All hypothesis tests were 2-sided with a familywise (primary analysis) or an unadjusted type I error rate of .05 (all secondary analyses). The primary intention-to-treat analysis modeled change in LDL-C level to months as a linear function of the intervention group and LDL-C level at baseline with adjustment by study site (Penn Medicine or Lancaster General Hospital) and used multiple imputation to account for missing data (16% of LDL-C levels at 12 months, balanced across groups).^20,39^ In the primary analysis, stage 1 tested whether the mean change in LDL-C level at 12 months differed between each intervention group and the control group using a Holm-Bonferroni correction for 3 contrasts. If differences were found, stage 2 would contrast each pair of interventions that differed from the control in stage 1.^40^

We designed the study to detect a 10-mg/dL or more mean difference change in LDL-C levels between the control and any intervention group—a value related to a clinically relevant reduction in ASCVD-related events.^35^ The assumed SD for the change in LDL-C level was 28 mg/dL. Assuming a 14.5% loss to follow-up for change in LDL-C levels, simulations indicated 187 participants in each group (n = 748 total) would provide more than 80% power to detect a 10-mg/dL or more difference between any intervention group and the control group, and in the event of progressing to stage 2, 82% power to detect a difference of 5 mg/dL between at least 1 pair of the intervention groups. We conducted multiple secondary and sensitivity analyses (eMethods 6 in Supplement 2). In an exploratory analysis, we also describe spillover effects of study interventions on changes in HbA_1c_ and systolic blood pressure levels collected from the electronic medical record in the 6-month period before randomization and at 9 to 15 months (eMethods 1 in Supplement 2).

Simulations for calculating sample size and analysis of the association between measured adherence and change in LDL-C levels were performed in R, version 2.5.1 (R Foundation for Statistical Computing). All other analyses were conducted using SAS version 9.4 (SAS Institute Inc). x

## Results

Figure 1 displays study enrollment and group assignment for the 764 participants; 656 (85.9%) were recruited from Penn Medicine and 108 (14.1%) were recruited from Lancaster General Hospital. Prerandomization invitation, screening, and exclusion numbers are displayed in eFigure 1 in Supplement 2. Participant characteristics were well balanced across study groups (Table 1; eTable 1 in Supplement 2). Mean (SD) age of the participants was 62.4 (10.0) years, 390 of 762 were women (51.2%), 319 of 759 (42.0%) were of races other than White, 538 of 762 (70.6%) had attended at least some college, and 413 of 762 (54.2%) were married or with a partner. Of the total population of 764 individuals, 310 (40.6%) had diabetes and 298 (39.0%) had hypertension. Compared with Penn Medicine, Lancaster General Hospital participants were almost exclusively of White race, had less education, were less likely to have diabetes, and were more reluctant to divulge their income (eTable 2 in Supplement 2).

**Table 1.** Baseline Participant Characteristics

Characteristic	Group, No. (%)				
	Total (N = 764)	Control (n = 190)	Process (n = 194)	Outcomes (n = 187)	Process plus outcomes (n = 193)
Age, mean (SD), y	62.4 (10.0)	62.4 (10.0)	63.1 (9.7)	62.5 (9.5)	61.7 (10.8)
Sex^a^					
Female	390 (51.2)	99 (52.1)	98 (50.8)	94 (50.3)	99 (51.6)
Male	372 (48.8)	91 (47.9)	95 (49.2)	93 (49.7)	93 (48.4)
Race^a^					
White	440 (58.0)	102 (53.7)	117 (60.9)	109 (58.6)	112 (58.6)
Black	271 (35.7)	74 (38.9)	68 (35.4)	61 (32.8)	68 (35.6)
Other^b^	48 (6.3)	14 (7.4)	7 (3.6)	16 (8.6)	11 (5.8)
Ethnicity^a^					
Hispanic or Latino	19 (2.5)	5 (2.7)	6 (3.1)	8 (4.3)	0
Not Hispanic or Latino	737 (97.5)	183 (97.3)	187 (96.9)	179 (95.7)	188 (100.0)
Educational level^a^					
High school or less	224 (29.4)	53 (27.9)	58 (29.9)	53 (28.5)	60 (31.3)
Some college	208 (27.3)	59 (31.1)	46 (23.7)	53 (28.5)	50 (26.0)
College degree	330 (43.3)	78 (41.1)	90 (46.4)	80 (43.0)	82 (42.7)
Income before taxes, $					
<50 000	303 (39.7)	77 (40.5)	74 (38.1)	75 (40.1)	77 (39.9)
≥50 000	338 (44.2)	81 (42.6)	88 (45.4)	83 (44.4)	86 (44.6)
Did not wish to answer^c^	123 (16.1)	32 (16.8)	32 (16.5)	29 (15.5)	30 (15.5)
Marital status^a^					
Single	184 (24.1)	57 (30.0)	46 (23.7)	42 (22.5)	39 (20.4)
Married/unmarried partner	413 (54.2)	103 (54.2)	102 (52.6)	97 (51.9)	111 (58.1)
Divorced/widowed	165 (21.7)	30 (15.8)	46 (23.7)	48 (25.7)	41 (21.5)
Smoke >5 cigarettes/d	62 (8.1)	17 (8.9)	10 (5.2)	12 (6.4)	23 (11.9)
Household size, mean (SD)	2.3 (1.2)	2.3 (1.2)	2.3 (1.2)	2.3 (1.1)	2.4 (1.4)
Recruitment site					
Penn Medicine	656 (85.9)	163 (85.8)	165 (85.1)	162 (86.6)	166 (86.0)
Lancaster General Hospital	108 (14.1)	27 (14.2)	29 (14.9)	25 (13.4)	27 (14.0)
Diagnosed with diabetes	310 (40.6)	68 (35.8)	78 (40.2)	79 (42.2)	85 (44.0)
Diagnosed with ASCVD	298 (39.0)	81 (42.6)	74 (38.1)	74 (39.6)	69 (35.8)
Baseline LDL-C, mean (SD), mg/dL	138.8 (37.6)	136.8 (34.2)	141.3 (39.0)	136.9 (36.4)	139.9 (40.5)

Abbreviations: ASCVD, atherosclerotic cardiovascular disease; LDL-C, low-density lipoprotein cholesterol.

SI conversion factor: To convert LDL-C to millimoles per liter, multiply by 0.0259.

^a^ Values may not sum to the total number of participants due to missing data at baseline.

^b^ The Other category comprises participants who specified American Indian or Alaska Native, Asian, Native Hawaiian or Other Pacific Islander, or chose Other with the option to give a different answer than the choices listed above.

^c^ Participants were asked to provide their annual pretax household income in $10 000 intervals. Those who did not wish to answer the question were asked if they were willing to share whether their income was greater or less than $50 000. Do not wish to answer includes participants unwilling to answer both questions.

**Figure 2.**
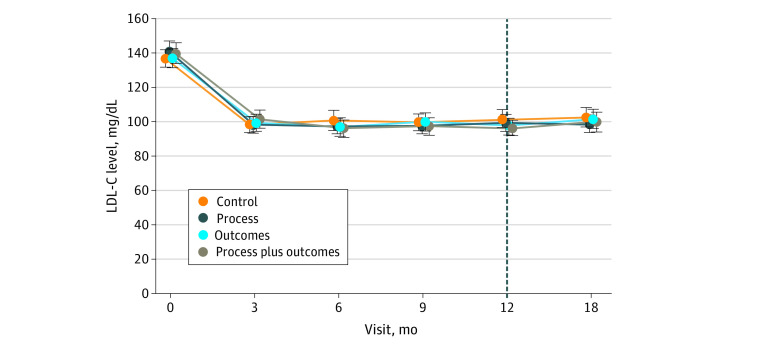
Mean Low-Density Lipoprotein Cholesterol (LDL-C) Levels by Visit and Intervention Group Error bars indicate 95% CIs. SI Conversion factor: To convert LDL-C to millimoles per liter, multiply by 0.0259.

The primary outcome at 12 months was measured in 644 participants (84.3%) and analyzed in 764 participants (Figure 1). Figure 2 shows mean LDL-C levels over time. At baseline, mean (SD) LDL-C level was 138.8 (37.6) mg/dL. After adjusting for baseline levels of LDL-C and site, mean 12-month LDL-C level reductions by group were substantial: −36.9 mg/dL (95% CI, −42.0 to −31.9 mg/dL) among participants in the control group, −40.0 mg/dL (−44.7 to −35.4 mg/dL) in the process group, −41.6 mg/dL (−46.3 to −37.0 mg/dL) in the outcomes group, and −42.8 mg/dL (−47.4 to −38.1 mg/dL) in the process plus outcomes group. Specifically, compared with the control group, the intervention arms had additional small reductions in LDL-C: −3.1 mg/dL (95% CI −9.9 to 3.7 mg/dL; adjusted *P* = .37) for the process group; −4.7 mg/dL (95% CI −11.4 to 2.0 mg/dL; adjusted *P* = .34) for the outcomes group; and −5.8 mg/dl (95% CI −12.4 to 0.7 mg/dl; adjusted *P* = .24) in the process plus outcomes group (Table 2). Because none of the intervention groups differed statistically significantly from the control group in this stage of the analysis, we did not move to stage 2 of the analysis; thus, no further pairwise comparisons were made. eTable 3 in Supplement 2 compares the characteristics of participants who did and did not complete the 12-month LDL-C level measurement.

**Table 2.** Primary Outcome of Change in LDL-C From Baseline to Completion of Intervention at 12 Months

Outcome	Control group	Process group	Outcomes group	Process plus outcomes group
Change in LDL-C^a^	−36.9 (−42.0 to −31.9)	−40.0 (−44.7 to −35.4)	−41.6 (−46.3 to −37.0)	−42.8 (−47.4 to −38.1)
Difference in change vs control^a^	NA	−3.1 (−9.9 to 3.7)	−4.7 (−11.4 to 2.0)	−5.8 (−12.4 to 0.7)
*P* value	NA	.37	.17	.08
Adjusted *P* value^b^	NA	.37	.34	.24

Abbreviations: LDL-C, low-density lipoprotein cholesterol; NA, not applicable.

SI conversion factor: To convert LDL-C to millimoles per liter, multiply by 0.0259.

^a^ Mean (95% CI) change from baseline from a linear model adjusted for baseline LDL-C and site. Results are reported for Penn Medicine at mean baseline LDL-C. Mean LDL-C at 12 months for Lancaster General Hospital participants was 0.8 mg/dL higher than for Penn Medicine participants. Multiple imputation was used to address incomplete data.

^b^ Holm-Bonferroni–adjusted *P* values based on 3 contrasts of each group with control.

Mean change in LDL-C levels in the intervention groups at 18 months (6 months after financial incentives ended and after the primary outcome) differed from the control group by less than 5 mg/dL (eTable 4 in Supplement 2). eFigure 2 in Supplement 2 shows change in LDL-C levels in 6 prespecified subgroups. Compared with the control group, the process plus outcomes group showed noticeable reductions in LDL-C levels vs control by 12 months in the following subgroups: baseline LDL-C levels greater than 160 mg/dL (n = 150; −100.4 vs −76.4 mg/dL; *P* = .002), ASCVD diagnosis (n = 281; −51.7 vs −36.4 mg/dL; *P* = .02), Black race (n = 258; −44.3 vs −30.9 mg/dL; *P* = .001), annual income less than $50 000 (n = 286; −44.2 vs −27.8 mg/dL; *P* = .01), and women (n = 375; −40.0 vs −34.0 mg/dL; *P* = .04). Reductions in LDL-C levels were also evident for the subgroup with ASCVD in the process group (n = 281; −44.4 vs −36.4 mg/dL; *P* = .03), for Black individuals in the process (n = 258; −38.0 vs −30.9 mg/dL; *P* = .02) and outcomes (n = 258; −43.7 vs −30.9 mg/dL; *P* = .047) groups, and for individuals with annual income less than $50 000 in the outcomes group (n = 286; −42.2 vs −27.8 mg/dL; *P* = .047).

Sensitivity analyses, including a complete case analysis, had little influence on the results (eTable 5, eTable 6, eFigure 3 in Supplement 2). A post hoc analysis of the outcome of changes in LDL-C levels between 3 and 12 months showed modest changes in mean LDL-C levels in all groups (eTable 7 in Supplement 2).

Despite small differences in change in LDL-C levels vs control, the 2 intervention groups with incentives for daily statin adherence (process and process plus outcomes) exhibited considerably higher rates of adherence measured with smart pill bottles. Measured adherence across 12 months was 70% (95% CI, 66%-74%) in both the control and outcomes groups compared with 83% in the process group (95% CI, 79%-87%) and 80% (95% CI, 76%-84%) in the process plus outcome group (both *P* < .001 vs control) (eFigure 4 and eTable 8 in Supplement 2).

During the 12-month intervention, median per-participant incentive payments were $445 (interquartile range, $365-$535) for process, $252 (interquartile range, $126-$504) for outcomes, and $366 (interquartile range, $274-$450) for process plus outcomes. eTable 9 in Supplement 2 lists adverse events; none were adjudicated as probably associated with study participation.

**Figure 3.**
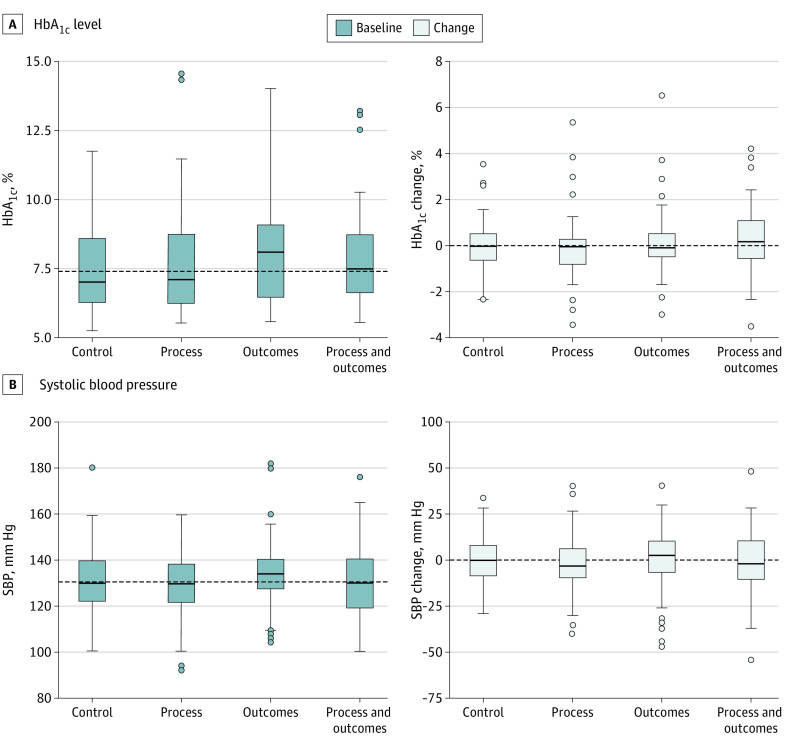
Spillover Analyses of Hemoglobin A_1c_ (HbA_1c_) Level and Systolic Blood Pressure by Intervention Group The horizontal line in the boxes indicates the median. The upper and lower ends of the boxes are 75th and 25th percentiles. The upper and lower whiskers are 1.5 times the interval range. The circles represent outliers. SI conversion factor: To convert HbA_1c_ to proportion of total hemoglobin, multiply by 0.01.

Overall, 458 participants with hypertension (87%) and 177 of those with diabetes (68%) were included in analyses of spillover effects on blood pressure and HbA_1c_ level (eFigure 5 in Supplement 2). The numbers of patients eligible for these analyses were similar across groups, as were baseline levels of HbA_1c_ and systolic blood pressure (Figure 3). The median change comparing baseline with 1-year measurements was near 0 for both HbA_1c_ level and systolic blood pressure in each group and the 95% CI on the mean always included 0 (Figure 3).

## Discussion

Nonadherence to effective medications is a major barrier to reducing mortality and morbidity among patients at risk for ASCVD. Financial incentives are candidate approaches to overcome present bias, inattention, low engagement with health, and other barriers to adherence. This trial tested a important contrast in the delivery of financial incentives: rewarding a process- vs an outcome-based incentive in the setting of a daily medication for cholesterol level control. We randomized 764 statin users and compared control with financial incentives for daily statin adherence, quarterly improvement in LDL-C level, or a combination of both. Overall, all 4 groups experienced a substantial decrease in LDL-C level, and no incentive produced a greater reduction in LDL-C level in the intervention groups than the control group. Taken together with other research on behavior change, these results suggest that effective interventions might need to combine financial incentives with other support, such as guidance and coaching, to help patients develop customized solutions to the particular obstacles they face.^20,27,41,42^

Health insurance companies, government agencies, and most employers have deployed financial incentives to promote healthy behaviors.^43,44^ Such programs have proliferated despite uncertainty about how to structure incentives effectively.^38^ Incentive theories from economics offer rationales for both process-of-care incentives and incentives directed at outcomes.^45^ Daily process incentives for medication adherence provide tangible, immediate feedback and could overcome human tendencies toward present bias. Social cognitive theory further suggests that a successful process incentive could increase an individual’s self-efficacy around medication adherence, helping to form a durable habit.^28,30,31,46^ Yet, the need to monitor process introduces challenges.^47^ We used smart pill bottles to reward adherence in real time, but the need for patients to integrate the bottles into their routines could have posed a challenge. Pill bottles may malfunction and design flaws might discourage patients.

Outcomes-based incentives offer an alternative by allowing individuals to improve their behavior in whatever manner they prefer or deem most efficient. For example, an individual receiving statin therapy might decide that keeping their pills in a prominent location works better than incentives tied to a smart device or that it is preferable to spend time exercising rather than managing medications.^48^ Furthermore, because the goal is to prevent myocardial infarction or death, improvement in outcomes such as LDL-C level is 1 step closer in the causal pathway to meaningful clinical events than is medication adherence.

This study, a direct comparison between process-based vs outcome-based incentives for medication adherence, found no evidence that, on average, patient-directed financial incentives of either kind improved LDL-C levels more than control.^20,41,42^ We did, however, observe significant treatment effects in secondary analyses of subgroups defined by sex, Black race, baseline LDL-C level, and elevated risk of ASCVD. These effects may inform the design of future research. Qualitative studies may help to determine whether and why incentives might be perceived differently in these groups. Future adherence studies may target these groups or anticipate greater efficacy among them.

Financial incentives may help patients focus on the importance of statins and LDL-C levels, but clearly, incentives were insufficient in this trial and the results raise the question whether present bias is the key barrier to the formation of healthy habits. Patients may also need support for diverse personal barriers that they may face, such as challenging living conditions. Future studies might combine incentives with interventions, such as motivational interviewing, for patients who feel that bad health is inevitable or who struggle to change their routines.^49^

Our results provide other lessons for future study design. Patients commonly have multiple comorbidities and behavioral challenges, such as a patient with obesity and diabetes who would benefit from adherence to statins and insulin, as well as better diet and weight management. Using electronic medical record data, we were able to examine the possibility that financial incentives focused on statins and LDL-C levels affected other health behaviors. The lack of any substantial differences in systolic blood pressure or HbA_1c_ level during the trial, across all groups, alleviates concerns that focusing attention on 1 health behavior may worsen other health outcomes.

### Limitations

This study has limitations. The trial outcome, LDL-C level, was simple to measure, directly connected to medication adherence, and not easily susceptible to bias, but LDL-C level is a surrogate outcome. Although there was no significant difference in the primary outcome between study groups, participants in all groups experienced a substantial improvement in LDL-C levels. We believe the best explanation is that the baseline increased LDL-C levels were detected in usual care and that, independent of trial participation, clinicians may have intensified participants’ statin therapy and encouraged other behavior changes; we were not able to gather robust information on changes in statin medications, diet, or exercise. Reversion to the mean is another possible explanation. In an earlier trial in the same population, the possibility that providing smart bottles alone was responsible for the LDL-C level improvement was excluded.^20^ A post hoc analysis of changes in LDL-C levels between 3 and 12 months also suggested the possibility that the effect of interventions may change over time, but the study was not designed to detect time-dependent effects. Another limitation is that the study was conducted primarily in an academic health system, which could limit generalizability. It is possible that financial incentives could be more effective at improving adherence and LDL-C levels among patients in other settings.^50^ In addition, adherence measured through smart pill bottles was significantly higher in the process and process plus outcomes groups, which incentivized use of the bottles. Although there may have been true differences in adherence across groups, despite no significant differences in the primary outcome, it is also plausible that the process incentive participants may have shifted from using their regular pill boxes to using the smart pill bottles.

## Conclusions

The results of this randomized clinical trial indicate that patient-directed financial incentives, whether targeting process or outcome, are ineffective. The interventions did not significantly improve LDL-C levels in patients with increased ASCVD risk.

## Notes

Supplement 1.
Trial Protocol

Supplement 2. **eMethods 1.** Outcome Ascertainment **eMethods 2.** Inclusion and Exclusion Criteria **eFigure 1.** Prerandomization Invitation, Screening, and Exclusion **eMethods 3.** List of Baseline Assessments **eMethods 4.** Smart Pill Bottles Use in the Trial **eMethods 5.** Methods Related to Administration of Financial Incentives **eMethods 6.** Methods Related to Secondary and Sensitivity Analyses **eTable 1.** Adherence Scores for Participants Enrolled in the Trial, by Study Group **eTable 2.** Baseline Characteristics of Participants Enrolled at Penn Medicine Sites Versus Lancaster General Hospital Sites **eTable 3.** Characteristics of Participants Who Did and Did Not Complete the 12-Month LDL-C Measurement **eTable 4.** Secondary Outcome of Change in LDL-C From Baseline to 18 Months (mg/dL) **eFigure 2.** Subgroup Analyses of the Primary Outcome of Change in LDL-C (mg/dL) From Baseline to 12 Months Using a Linear Mixed-Effects Model **eTable 5.** Secondary Complete Case Analysis for Outcome of Change in LDL-C (mg/dL) From Baseline to 12 Months Using the Same Model as Reported for the Primary Analysis **eFigure 3.** Secondary Analysis of Data From All Visits During the Intervention (3, 6, 9, and 12 Months) **eTable 6.** Sensitivity of Primary Results to Different Models; Change in LDL-C (mg/dL) From Baseline to 12 Months Using a Linear Model With Adjustment for Different Baseline Covariates **eTable 7.** Change in LDL-C (mg/dL) From 3-Month Visit to 12-Month Visit **eFigure 4.** Secondary Outcome of Mean Weekly Measured Statin Adherence, by Intervention Arm **eTable 8.** Secondary Outcome of Mean Weekly Measured Statin Adherence, by Intervention Arm **eTable 9.** Summary of Adverse Events **eFigure 5.** Spillover Analysis Flowchart of Penn Medicine Patients With Hypertension and Diabetes Plus Usual Care Data for Hemoglobin A1c and Systolic Blood Pressure, Respectively, in the Prerandomization Period (up to 6 Months Prior) and Later Period (9-15 Months Postrandomization)

Supplement 3.
Data Sharing Statement
