# Supplementary material for: Effect of Financial Incentives for Process, Outcomes, or Both on Cholesterol Level Change: A Randomized Clinical Trial
Source: JAMA Netw Open. 2021 Oct 4;4(10):e2121908. doi: 10.1001/jamanetworkopen.2021.21908 (PMC8491106; doi:10.1001/jamanetworkopen.2021.21908)
Supplement: Supplement 1. — Trial Protocol [file jamanetwopen-e2121908-s001.pdf]

Modification

Basic Info

Confirmation Number:	**cjchgajg**
Protocol Number:	**818440**
Created By:	**GATTO, DANA**
Principal Investigator:	**REESE, PETER**
Protocol Title:	**Comparative Effectiveness of Process and Outcomes Incentives for Lipid Management**
Short Title:	**Process Versus Outcomes Incentives for Lipid Management**
Protocol Description:	**In a 4-arm, randomized controlled trial, we propose to evaluate the relative effectiveness and cost-effectiveness of improving cholesterol levels among participants who are at high risk of CVD and who have elevated LDL cholesterol levels by testing process versus outcomes financial incentives. Participants will use electronic pill bottles that continuously monitor statin adherence. The primary outcome will be change in LDL cholesterol over 12 months.**
Submission Type:	**Biomedical Research**
Application Type:	**EXPEDITED Category 2**

PennERA Protocol Status

Approved (No CR)

Resubmission*
No

Are you submitting a Modification to this protocol?*

Yes

Current Status of Study

Study Status

Closed to subject enrollment (remains active)

If study is currently in progress, please enter the following

Number of subjects enrolled at Penn since the study was initiated

0

Actual enrollment at participating centers

0

If study is closed to further enrollment, please enter the following

Number of subjects in therapy or intervention

0

Number of subjects in long-term follow-up only

0

IRB Determination
If the change represents more than minimal risk to subjects, it must be reviewed and approved by the IRB at a convened meeting. For a modification to be considered more than minimal risk, the proposed change would increase the risk of discomfort or decrease benefit. The IRB must review and approve the proposed change at a convened meeting before the change can be implemented unless the change is necessary to eliminate an immediate hazard to the research participants. In the case of a change implemented to eliminate an immediate hazard to participants, the IRB will review the change to determine that it is consistent with ensuring the participant′s continued welfare. Examples: Convened Board Increase in target enrollment for investigator initiated research or potential Phase I research Expanding inclusion or removing exclusion criteria where the new population may be at increased risk Revised risk information with active participants Minor risk revisions that may affect a subject′s willingness to continue to participate Expedited Review Increase in target enrollment at Penn where overall enrollment target is not exceeded or potentially sponsored research Expanding inclusion or removing exclusion where the new population has the same expected risk as the previous, based on similarities of condition Revised risk information with subjects in long-term follow-up Minor risk revisions with no subjects enrolled to date
Expedited Review

Modification Summary
Please describe any required modification to the protocol. If you are using this form to submit an exception or report a deviation, enter 'N/A' in the box below.
Dear IRB Administrator, On behalf of Dr. Peter Reese, we are submitting a modification to add Adam Mussell to our IRB protocol. Adam is replacing our current project manager who is leaving the university. Adam is a current project manager on multiple studies at Penn and his Ctit training is up to date. We would also like to take this time to remove of the following staff as the no longer work on this project.

Risk / Benefit
Does this amendment alter the Risk/Benefit profile of the study?
No

Change in Consent
Has there been a change in the consent documents?
No

If YES, please choose from the options below regarding re-consenting

Deviations

Are you reporting a deviation to this protocol?*

No

Exceptions

Are you reporting an exception to this protocol?*

No

Protocol Details

Resubmission*
Yes

Hospital Sites
Will any research activities and/or services be conducted at a Penn Medicine affiliated hospital site?
No

Study Personnel

Principal Investigator

Name:	**REESE, PETER**
Dept / School / Div:	**4259 - DM-Renal-Electrolyte and Hypertension**
Campus Address Mail Code	
Address:	**917 Blockley Hall CCEB<br>423 Guardian Drive**
City State Zip:	**Philadelphia PA 19104-4865**
Phone:	**215-900-3782**
Fax:	**215-615-0349**
Pager:	
Email:	**Peter.Reese@uphs.upenn.edu**
HS Training Completed:	**Yes**
Training Expiration Date:	**09/15/2014**
Name of course completed :	**CITI Protection of Human Subjects Research Training - ORA**

Study Contacts

Name:	**PAGNOTTI, DAVID R**
Dept / School / Div:	**10599 - ME-Division of Health Policy**
Campus Address Mail Code	
Address:	
City State Zip:	
Phone:	**215-573-2770**
Fax:	
Pager:	
Email:	**davidrp@pennmedicine.upenn.edu**
HS Training Completed:	**Yes**
Training Expiration Date:	**09/13/2018**
Name of course completed :	**CITI Protection of Human Subjects Research Training - ORA**

Other Investigator

Name:	**VOLPP, KEVIN G**
Dept / School / Div:	**10599 - ME-Division of Health Policy**
Campus Address Mail Code	**6021**
Address:	**BLOCKLEY HALL<br>423 GUARDIAN DR**
City State Zip:	**PHILADELPHIA PA 19104-6021**
Phone:	**215-573-0270**
Fax:	**-**
Pager:	
Email:	**volpp70@wharton.upenn.edu**
HS Training Completed:	**Yes**
Training Expiration Date:	**12/10/2015**
Name of course completed :	**CITI Protection of Human Subjects Research Training - ORA**

Responsible Org (Department/School/Division):

4259 - DM-Renal-Electrolyte and Hypertension

Key Study Personnel

Name:	**HA, YOONHEE P**
Department/School/Division:	**Health System**
HS Training Completed:	**Yes**
Training Expiration Date:	**12/24/2019**
Name of course completed:	**CITI Protection of Human Subjects Research Training - ORA**

Name:	**PAGNOTTI, DAVID R**
Department/School/Division:	**ME-Division of Health Policy**
HS Training Completed:	**Yes**
Training Expiration Date:	**09/13/2018**
Name of course completed:	**CITI Protection of Human Subjects Research Training - ORA**

Name:	**BARANKAY, IWAN**
Department/School/Division:	**Management**
HS Training Completed:	**Yes**
Training Expiration Date:	**08/29/2015**
Name of course completed:	**CITI Protection of Human Subjects Research Training - ORA**

Name:	**YAN, JIALI**
Department/School/Division:	**DM-General Internal Medicine**
HS Training Completed:	**Yes**
Training Expiration Date:	**06/08/2020**
Name of course completed:	**CITI Protection of Human Subjects Research Training - ORA**

Name:	**PUTT, MARY**
Department/School/Division:	**BE-Biostatistics Division**
HS Training Completed:	**Yes**
Training Expiration Date:	**10/22/2016**
Name of course completed:	**CITI Protection of Human Subjects Research Training - ORA**

Name:	**HUANG, QIAN**
Department/School/Division:	**DM-General Internal Medicine**
HS Training Completed:	**Yes**
Training Expiration Date:	**10/20/2017**
Name of course completed:	**CITI Protection of Human Subjects Research Training - ORA**

Name:	**SOFFER, DANIEL**
Department/School/Division:	**DM-Palliative and Advanced Illness Research Center**
HS Training Completed:	**Yes**
Training Expiration Date:	**08/26/2019**
Name of course completed:	**CITI Protection of Human Subjects Research Training - ORA**

Name:	**PHILLIPS, CAITLIN**
Department/School/Division:	**Health System**
HS Training Completed:	**Yes**
Training Expiration Date:	**10/24/2019**
Name of course completed:	**CITI Protection of Human Subjects Research Training - ORA**

Name:	**MUSSELL, ADAM S**
Department/School/Division:	**DM-Renal-Electrolyte and Hypertension**
HS Training Completed:	**No**
Training Expiration Date:	
Name of course completed:	

Disclosure of Significant Financial Interests*
Does any person who is responsible for the design, conduct, or reporting of this research protocol have a **FINANCIAL INTEREST**?
No

Penn Intellectual Property*
To the best of the Principal Investigator's knowledge, does this protocol involve the testing, development or evaluation of a drug, device, product, or other type of intellectual property (IP) that is owned by or assigned to the University of Pennsylvania?
No

Certification
I have reviewed the *Financial Disclosure and Presumptively Prohibited Conflicts for Faculty Participating in Clinical Trials* and the *Financial Disclosure Policy for Research and Sponsored Projects* with all persons who are responsible for the design, conduct, or reporting of this research; and all required Disclosures have been attached to this application.
Yes

Biomedical Research

Clinical Trial*
Is this a clinical trial?
Yes

If Yes, please be aware that for each clinical trial conducted or supported by a Federal department or agency, one IRB-approved informed consent form used to enroll subjects must be posted by the awardee or the Federal department or agency component conducting the trial on a publicly available Federal Web site that will be established as a repository for such informed consent forms.

Investigator Initiated Trial*
Is this an investigator initiated trial?
Yes

If Yes, please be aware that the investigator may be required to create and manage a record of this trial in https://clinicaltrials.gov.

Drugs or Devices*
Does this research study involve Drugs or Devices?
No

IND Exemption

For studies that fall under an IND exemption, please provide the number below

For studies including IND or IDE's, please provide the number(s) below

IDE Review*
NOTE: For research involving investigational devices, you are required to review the guidance on Managing Research Device Inventory. Consult the Penn Manual for Clinical Research: https://www.med.upenn.edu/pennmanual/secure/investigational-product-management-at-sites-not-using-investigational-drug-services-(ids).html Please check the box Yes if you have reviewed the guidance.
Yes

Research Device Management*
Please indicate how research device(s) will be managed.
Not Applicable (no investigational devices)

Drug, Herbal Product or Other Chemical Element Management *
Please indicate how drugs, herbal products or other chemical entities will be managed.
Not Applicable (no drugs, herbal products or other chemical entities)

Radiation Exposure*
Are research subjects receiving any radiation exposure (e.g. X-rays, CT, Fluoroscopy, DEXA, pQCT, FDG, Tc-99m, etc.) that they would not receive if they were not enrolled in this protocol?
No

Gene Transfer*
Does this research involve gene transfer (including all vectors) to human subjects?
No

Human Source Material*
Does this research include collection or use of human source material (i.e., human blood, blood products, tissues or body fluids)?
Yes

CACTIS and CT Studies*
Does the research involve Center for Advanced Computed Tomography Imaging Services (CACTIS)

and CT studies that research subjects would not receive if they were not part of this protocol?
No

CAMRIS and MRI Studies*
Does the research involve Center for Advanced Magnetic Resonance Imaging and Spectroscopy (CAMRIS) and MRI studies that research subjects would not receive if they were not part of this protocol?
No

Investigational Agent or Device within the Operating Room*
Does the research project involve the use of an investigational agent or device within the Operating Room?
No

Cancer Related research not being conducted by an NCI cooperative group*
Does this protocol involve cancer-related studies in any of the following categories?
No

Processing of Materials*
Will the research involve processing (such as over encapsulating, or compounding)?
No

In-House Manufacturing of Materials*
Will the research involve processing (such as over encapsulating, or compounding)?
No

Medical Information Disclosure*
Does the research proposal involve the use and disclosure of research subject's medical information for research purposes?
Yes

If the answer is YES, indicate which items is is provided with this submission:

Modified research informed consent document that incorporates HIPAA requirements

CTRC Resources*
Does the research involve CTRC resources?
No

Pathology and Laboratory Medicine Resources*
Will samples be collected by hospital phlebotomy and/or processed or analyzed by any of the clinical laboratories of the University of Pennsylvania Health System?
No

Research Involves Apheresis, Cell Collection, and/or Blood Product Collection*
Does this research involve collection of blood products in the Penn Donor Center and/or the use of apheresis for treatment or collection of cells or other blood components?
No

Research involving blood transfusion or drug infusions*
Will your research involve blood transfusion or infusion of study drug in 3 Ravdin Apheresis Unit for research purposes?
No

Trial in Radiation Oncology
Is this research a prospective trial being done in Radiation Oncology, and if so, has this protocol been approved by the Radiation Oncology Protocol committee?
No

Study in Radiation Oncology
Is this research a retrospective study being done in Radiation Oncology, and if so, has this project been reviewed by the Radiation Oncology Clinical Research Group?

No

Use of UPHS services*
Does your study require the use of University of Pennsylvania Health System (UPHS) services, tests or procedures*, whether considered routine care or strictly for research purposes?
No

Primary Focus*
Sociobehavioral (i.e. observational or interventional)

Protocol Interventions

x	**Sociobehavioral (i.e. cognitive or behavioral therapy)**
	**Drug**
	**Device - therapeutic**
	**Device - diagnostic (assessing a device for sensitivity or specificity in disease diagnosis)**
	**Surgical**
x	**Diagnostic test/procedure (research-related diagnostic test or procedure)**
	**Obtaining human tissue for basic research or biospecimen bank**
x	**Survey instrument**
	**None of the above**

The following documents are currently attached to this item:

There are no documents attached for this item.

Department budget code

None

Multi-Center Research

Penn as lead
1. Is this a multi-center study where Penn is serving as the Lead Site or the Penn PI is serving as the Lead Investigator?
Yes

Management of Information for Multi-Center Research
N/A

Penn irb of record
2. Is this a multi-center study where the Penn IRB will be asked to serve as the IRB of Record for other external study sites?
No

Other Sites

No other sites

Protocol

Abstract
Cardiovascular disease (CVD) is the leading cause of death in the United States (US) and costs the US healthcare system $500 billion annually. Statins, also known as HMG-CoA reductase inhibitors, have been shown to improve cholesterol and lower mortality from CVD in multiple clinical trials. The majority of patients with CVD or diseases with equivalent cardiovascular risk, such as diabetes, require a statin to achieve the low-density cholesterol (LDL) targets in clinical practice guidelines. Despite the substantial benefits and reasonable risks associated with statins, adherence is strikingly poor with adherence rates often about 50% within the year following a heart attack. Therefore, reducing CVD-related morbidity, mortality and health care costs will depend to a great degree on effective strategies to help patients improve medication adherence. Financial incentives have been shown to be effective at improving patient health behaviors including medication adherence in a wide variety of contexts. The delivery of such incentives has been enhanced by the recent development of new wireless technologies that facilitate the measurement of daily medication adherence and the provision of incentives in an automated fashion. However, the relative effectiveness of incentives based on process (e.g. statin adherence) versus outcome (e.g. improvements in LDL cholesterol) is unknown. The results will grow in policy relevance with the implementation of Section 2705 of the Affordable Care Act in 2014, which allows employers to utilize as much as 30% of premiums (50% if programs include smoking) for outcome-based incentives for metrics like LDL cholesterol. We propose a randomized controlled trial to evaluate the relative effectiveness and cost-effectiveness of improving cholesterol levels among participants who are at high risk of CVD (goal LDL 100 mg/dl) and who have elevated LDL cholesterol levels (100 mg/dl) by testing process versus outcomes financial incentives. Participants will use electronic pill bottles that continuously monitor statin adherence. The primary outcome will be change in LDL cholesterol over 12 months. LDL cholesterol will be measured again at 18 months, six months after the intervention has ended, to assess the durability of financial incentives on the outcome. The trial will have a 2 x 2 factorial design in which the 748 participants receive either: 1) sweepstakes-based financial incentives for statin adherence -- with participants eligible each day they take their medication correctly (process incentive group); 2) financial incentives for lowering LDL cholesterol by 10 mg/dl every 3 months (outcome incentive group); 3) a combination of daily sweepstakes incentives and incentives for lowering LDL cholesterol (process plus outcomes); or 4) usual care (the control). The study will achieve the following Specific Aims: 1) Assess the effectiveness of a process-based incentive on lowering LDL cholesterol; 2) Assess the effectiveness of an outcome-based incentive on lowering LDL cholesterol; 3) Assess the effectiveness of a combined process plus outcomes incentive on lowering LDL cholesterol; 4) Assess the cost-effectiveness of each of the interventions.

Objectives

Overall objectives
The study will achieve the following Specific Aims: 1) Assess the effectiveness of a process-based incentive on lowering LDL cholesterol; 2) Assess the effectiveness of an outcome-based incentive on lowering LDL cholesterol; 3) Assess the effectiveness of a combined process plus outcomes incentive on lowering LDL cholesterol; 4) Assess the cost-effectiveness of each of the interventions.

Primary outcome variable(s)
The primary outcome will be change in LDL cholesterol over 12 months.

Secondary outcome variable(s)
Secondary outcome variables include statin adherence assessed through Vitality GlowCaps or MedSignals, statin adherence assessed through the Medication Possession Ratio (MPR) and adherence to non-statin medications assessed through the Medication Possession Ratio.

Background
Cardiovascular disease (CVD) is the leading cause of death in the United States. CVD risk can be significantly reduced by treating dyslipidemia. Statins, also known as HMG-CoA reductase inhibitors, have been shown to improve cholesterol and lower mortality from CVD in multiple clinical trials.8,9 The majority of patients with CVD or diseases with equivalent cardiovascular risk, such as diabetes, require a statin to achieve the low-density cholesterol (LDL) targets in clinical practice guidelines.10 Despite the substantial benefits and reasonable risks associated with statins,2 adherence is strikingly poor.11-13 For example, one year after hospitalization for an acute coronary syndrome, nearly half of

patients prescribed statins stop taking them.14 Reducing CVD-related morbidity, mortality and health care costs will depend to a great degree on effective strategies to help patients improve medication adherence and other health behaviors.15 Financial incentives have been shown to be effective at improving patient health behaviors including medication adherence in a wide variety of contexts.16-24 The delivery of such incentives has been enhanced by the recent development of new wireless technologies that facilitate the measurement of daily medication adherence and the provision of incentives in an automated fashion.7 However, the relative effectiveness of incentives based on process (e.g. medication adherence) vs. outcome (e.g. improvements in LDL cholesterol) is unknown. Traditional economists would argue that incentivizing outcomes is likely to be more effective (each individual determines the most efficient path to a desired result) whereas behavioral economists would argue for incentivizing on process (which may be simpler, more tangible, and provides more frequent feedback). A rigorous experimental study that examines the relative effectiveness of equivalent value incentives based on process (statin adherence), outcomes (LDL cholesterol), or a combination of process and outcomes would provide an answer to a question of fundamental importance to the design of incentive programs. The results will grow in policy relevance with the implementation of Section 2705 of the Affordable Care Act in 2014, which allows employers to utilize as much as 30% (50% including smoking) of premiums for outcome-based incentives for metrics like LDL.6,25 A recent set of guidelines related to cholesterol management (Stone et. al, Circulation 2013) identified groups of patients at elevated risk of CVD that should be treated with statins but changed the orientation of clinical practice away from achieving particular LDL levels. The study DSMB determined that, since this study will enroll subjects at elevated risk for cardiovascular disease, it is reasonable to use changes in LDL as the primary outcome. . Improved adherence to statins will plausibly lead to reductions in LDL cholesterol; both improved statin adherence and lower LDL cholesterol could reduce CVD risk.

Literature Cited 1. Armitage J. The safety of statins in clinical practice. Lancet 2007;370:1781-90. 2. Baigent C, Keech A, Kearney PM, et al. Efficacy and safety of cholesterol-lowering treatment: prospective meta-analysis of data from 90,056 participants in 14 randomised trials of statins. Lancet 2005;366:1267-78. 3. Charles EC, Olson KL, Sandhoff BG, McClure DL, Merenich JA. Evaluation of cases of severe statin-related transaminitis within a large health maintenance organization. Am J Med 2005;118:618-24. 4. Graham DJ, Staffa JA, Shatin D, et al. Incidence of hospitalized rhabdomyolysis in patients treated with lipid-lowering drugs. JAMA 2004;292:2585-90. 5. A Trial of Behavioral Economic Interventions to Reduce Cardiovascular Disease (CVD) Risk. URL: http://www.clinicaltrials.gov/ct2/show/NCT01346189?term=RC4&rank=6. 6. Incentives for Nondiscriminatory Wellness Programs in Group Health Plans. Department of Health and Human Services. In: 45 CFR Parts 146 and 147: Federal Register, Vol 77, No. 227; November 26, 2012. 7. http://www.vitality.net. 8. Randomised trial of cholesterol lowering in 4444 patients with coronary heart disease: the Scandinavian Simvastatin Survival Study (4S). Lancet 1994;344:1383-9. 9. Cannon CP, Braunwald E, McCabe CH, et al. Intensive versus moderate lipid lowering with statins after acute coronary syndromes. N Engl J Med 2004;350:1495-504. 10. Third Report of the National Cholesterol Education Program (NCEP) Expert Panel on Detection, Evaluation, and Treatment of High Blood Cholesterol in Adults (Adult Treatment Panel III) final report. Circulation 2002;106:3143-421. 11. Shah ND, Dunlay SM, Ting HH, et al. Long-term medication adherence after myocardial infarction: experience of a community. Am J Med 2009;122:961 e7-13. 12. Ho PM, Magid DJ, Shetterly SM, et al. Medication nonadherence is associated with a broad range of adverse outcomes in patients with coronary artery disease. Am Heart J 2008;155:772-9. 13. Ho PM, Rumsfeld JS, Masoudi FA, et al. Effect of medication nonadherence on hospitalization and mortality among patients with diabetes mellitus. Arch Intern Med 2006;166:1836-41. 14. Jackevicius CA, Mamdani M, Tu JV. Adherence with statin therapy in elderly patients with and without acute coronary syndromes. JAMA 2002;288:462-7. 15. Loewenstein G, Brennan T, Volpp KG. Asymmetric paternalism to improve health behaviors. JAMA 2007;298:2415-7. 16. Volpp KG, Troxel AB, Pauly MV, et al. A randomized, controlled trial of financial incentives for smoking cessation. N Engl J Med 2009;360:699-709. 17. Volpp KG, John LK, Troxel AB, Norton L, Fassbender J, Loewenstein G. Financial incentive-based approaches for weight loss: a randomized trial. JAMA 2008;300:2631-7. 18. Jeffery RW, Forster JL, Snell MK. Promoting weight control at the worksite: a pilot program of self-motivation using payroll-based incentives. Prev Med 1985;14:187-94. 19. Forster JL, Jeffery RW, Sullivan S, Snell MK. A work-site weight control program using financial incentives collected through payroll deduction. J Occup Med 1985;27:804-8. 20. Higgins ST, Budney AJ, Bickel WK, Foerg FE, Donham R, Badger GJ. Incentives improve outcome in outpatient behavioral treatment of cocaine dependence. Arch Gen Psychiatry 1994;51:568-76. 21. Vandrey R, Bigelow GE, Stitzer ML. Contingency management in cocaine abusers: a dose-effect comparison of goods-based versus cash-based incentives. Exp Clin Psychopharmacol 2007;15:338-43. 22. Peirce JM, Petry NM, Stitzer ML, et al. Effects of lower-cost incentives on stimulant abstinence in methadone maintenance

treatment: a National Drug Abuse Treatment Clinical Trials Network study. Arch Gen Psychiatry 2006;63:201-8. 23. Charness G, Gneezy U. Incentives to Exercise. Econometrica 2009;77:909 - 31. 24. Petry NM, Alessi SM, Marx J, Austin M, Tardif M. Vouchers versus prizes: contingency management treatment of substance abusers in community settings. J Consult Clin Psychol 2005;73:1005-14. 25. Volpp KG, Asch DA, Galvin R, Loewenstein G. Redesigning employee health incentives--lessons from behavioral economics. N Engl J Med 2011;365:388-90.

Study Design

Phase*
Not applicable

Design
This 4-arm RCT will compare the relative effectiveness and cost-effectiveness of process versus outcome-based financial incentives in improving LDL cholesterol in patients who are at high risk for CVD and have been prescribed statins. Adherence in all groups will be measured using an electronic pill container as a recording device. The intervention will consist of an active phase of 12 months followed by 6 additional months of observation. Participants will be randomized evenly into one of 4 arms. The primary outcome will be change in LDL cholesterol from baseline to 12 months. All participants will have LDL cholesterol checked at baseline, 3, 6, 9, and 12 months during the interventions, and at 18 months (without incentives, in order to assess the sustainability of the effects achieved by the incentives). We selected cholesterol for this study of process versus outcomes because a change in LDL depends on sustained adherence to a statin. LDL cholesterol is easily, inexpensively and objectively measured. The impressive degree of risk reduction associated with LDL lowering is well established. The interventions will consist of financial incentives lasting 12 months for arms 1, 3, and usual care (no incentives) for arm 4. The net value of incentive payments are equivalent in arms 1, 2 and 3. Participants in Process (arm #1) and the Process plus Outcomes (arm #3) groups will be eligible for an adherence-based sweepstakes. The expected value of the daily sweepstakes payments will be $1.40 in the Process arm, and will be $0.70 in the Process with Outcomes arm. Thus, participants in arms 1 and 3 may benefit from daily engagement and the opportunity for multiple, short-term rewards in changing adherence behavior. At months 3, 6, 9, and 12 every participant in the Outcomes (arm #2) and the Process with Outcomes (arm #3) interventions will be eligible for a payment ($126 to arm #2; $63 to arm #3) if the participants LDL has improved by 10 mg/dl compared to the LDL at the end of the previous quarter. The daily expected value of these rewards per day will be equivalent to each of the other intervention arms. Study sites. Participants in the study will be employees of corporations with CVS-Caremark pharmacy benefits (see letter of support from Troyen Brennan) and Health System patients (Penn Medicine and Lancaster General Health (LGH)). The study will be run by UPENN investigators, who will communicate with participants through the Way to Health portal and by phone. Inclusion criteria. On further review of the recent AHA guidelines, we are changing our inclusion criteria to invite anyone with LDL190 (regardless of disease risk factors) as well as anyone with CAD with LDL100. The criteria includes: Individuals at high risk of a cardiac event, specifically one of the following: Individuals with clinical ASCVD (defined as diagnosis with myocardial infarction, stroke, or peripheral vascular disease) with an LDL greater than or equal to 100 mg/dl ; Individuals with Diabetes (between the ages of 40-75) with an LDL greater than or equal to 100 mg/dl; Individuals without clinical ASCVD or diabetes with LDLC with an LDL greater than or equal to 100 mg/dl and estimated 10-year ASCVD risk 7.5%; Individuals without clinical ASCVD or diabetes with LDL cholesterol 190 mg/dl A prescription filled for a statin medication within the last 12 months (derived from pharmacy records); Imperfect statin adherence level as defined by one of the following: Medication Possession Ratio (MPR) less than or equal to 80% A score 0 on the 8-item Morisky Medication Adherence Questionnaire completed during enrollment Exclusion criteria. Patients will be excluded if they 1) are younger than 18 years old; 2) have a contraindication to further statin use or have suffered statin side effects, such as myopathy; 3) will not or cannot give consent; 4) have a history of active or progressive liver disease are participating in another clinical trial with related aims, 6) have co-morbidities likely to lead to death within a short period (e.g. metastatic cancer), or 7) are prescribed by their doctor PCSK9 inhibitor injections coupled with statin therapy. Study Procedures Recruitment. All potentially eligible employees within the CVS-covered group will be contacted via existing communication channels through CVS. Potentially eligible employees will have evidence of diabetes medication use and/or CVD medication use in the CVS pharmacy database. These individuals will be sent letters (paper or email) describing the study and inviting them to enroll. Potentially eligible patients recruited from Penns Health system will be contacted by the study team via letter, email and phone calls. Baseline assessment

for participants. Interested employees/patients will have the option of entering data related to eligibility and their demographic and clinical characteristics through the Way to Health internet portal or by phone. The informed consent can be accessed on the study portal and signed electronically. The informed consent contains contact information for the study team if the participant wishes to go over the consent form with a team member by phone before electronically agreeing to participate by clicking the 'I want to participate' button at the end of the electronic consent form. If the patient agrees to complete the informed consent over the phone with the coordinator, the patient is allowing the coordinator to electronically agree to participate by clicking the 'I want to participate' button at the end of the electronic consent form. Each potential participant will visit a local participating laboratory (through the Quest national chain of commercial laboratories) to confirm that their LDL is greater than 100 mg/dl Approximately 3mL of blood will be drawn at each visit. All laboratory procedures will be paid for by the study. The enrollment interview and baseline labs will allow investigators to determine final eligibility for the study. The Quest corporation has 24 hour appointment scheduling and 2,000 lab locations; these locations are in every state as well as Puerto Rico. If the participant is from Penn Medicine, the study coordinator will order their labs in EPIC so the lab can be completed at their doctor's office. Our group has agreements in place with the Quest chain allowing research participants nationwide to have phlebotomy (blood draws). In the context of generous participation incentives (as will be provided in this study), the requirement of providing biological specimens at local laboratories has not been a barrier to program implementation. In another ongoing study, we have used Quest to enable 500 participants to get labs drawn at local laboratories. Randomization. Randomization will be performed through the Way to Health platform. Randomization will be stratified by employer (should more than one employer be involved to recruit a sufficient number of participants) and will use block randomization with variable block sizes. After reviewing the intake data and LDL, research staff will notify each participant of assignment using their preferred means of communication. All participants will receive an electronic pill container through the mail and will receive careful instructions about study procedures for their assigned arm. Throughout the study, coordinators will remain available to answer questions about the electronic pill container use and other study procedures. LDL Measurement Schedule. Participants will have six LDL laboratory checks, at months 0, 3, 6, 9, 12 and 18 months. To maximize the likelihood that these LDL measurements are completed, the Way to Health participant tracking system will remind the study coordinators when each participant is due to visit a lab. We will obtain extensive contact information including preferred mode of communication - from each participant and update it at each follow-up visit. Participants who miss scheduled LDL checks will receive four reminders. Patients will receive $50 after enrollment, initial blood work, and setting up the Glow Caps. A subset of participants will receive $100 after enrollment to test whether higher participation incentives increase recruitment rate. Participants will receive $50 for each subsequent lab check and $75 for the 12 month LDL check (the primary outcome). Administration of Process Incentives for Daily Adherence. The process financial incentive involves a daily sweepstakes for taking the statin medication. The sweepstakes has two components a high probability/low value reward as well as a low probability/high value reward. We will assign each participant in the Process (arm 1) and Process plus Outcome (arm 3) a two-digit number. Each day, the an electronic pill container will automatically upload the previous days data about each participants statin adherence via a wireless internet connection to the Way to Health database at UPENN. We will let each participant pick a two-digit sweepstakes number. Each day, Way to Health software will compare this number with a random two-digit number generated by Way to Health to determine eligibility for sweepstakes awards. For a participant who was adherent the previous day, if the two digit number matches exactly (probability of 1 in 100), the participant will win $50. When the two-digit number does not match, but either the first digit or second digit matches, the subject will win $5 (probability of 18 in 100). The expected value of this sweepstakes is $1.40/day such that total winnings over the 12-month trial for a participant with perfect adherence will have an average expected value of $511. An important feature of the sweepstakes is that each eligible participant will receive daily messages about the amount they won. Non-adherent participants will also receive daily feedback about whether they would have won if they had been adherent. This program incorporates key aspects of optimal design, including objective and reliable confirmation of behavior change at frequent intervals, large potential payments to reinforce the target behavior, frequent reinforcement using high probability/low value awards, and the use of anticipated regret, a powerful motivator. We set the expected value of the sweepstakes at $1.40/day based on our previous success in significantly affecting weight loss and medication adherence with this design. In addition, there is evidence that a patient with established CVD has health expenditures ranging from $4,500 to as high as $30,000 per year, suggesting that $1.40/day incentives if effective, could be cost-effective due to the potential savings in avoiding future CVD events. Administration of Outcome Incentives for LDL. Participants in the outcomes arms (arms #2 and #3) will receive payments if their

LDL decreases at least 10 mg/dl in the 3 months since the last visit. Over a year, these payments will be equivalent in magnitude to those in the process arm. The 3-month payment for the Outcome arm (arm #2) will be $126, while the payment for the Process with Outcomes Incentive arm (arm #3) will be half this value, or $63. Notably, if a participant in the Outcomes arm or the Process plus Outcomes arm achieves a reduction in LDL 20 mg/dl during one quarter of the trial and that reduction persists during the next quarter, the participant would be eligible for the outcome incentive at the end of both quarters, even if no further reduction in LDL took place during the second quarter. For instance, if a patient in the Outcome arm had an LDL decrease from 150 to 130 mg/dl between baseline and month 3, and also had an LDL of 130 mg/dl at month 6, that person would receive the incentive payment at month 3 and month 6. Participants in the intervention arms will be informed if they fail to meet adherence or LDL goals, and thereby are not eligible for payments. Providing this feedback may also improve adherence by taking advantage of anticipated regret. Outcomes. LDL cholesterol. The primary outcome will be change in LDL cholesterol between baseline (study enrollment) and 12 months. We will also measure LDL cholesterol at 18 months three months after interventions have ended. The 18 month measurement will allow us to ascertain the durability of lipid-lowering effects attributable to financial incentives. LDL cholesterol is a strong risk factor for CVD, and reductions in LDL for instance, through statin treatment lead to meaningful reductions in the risk of cardiovascular events. LDL cholesterol is easily assessed through a blood test. LDL can be calculated indirectly from other measured lipids if the patient is fasting, or measured directly. We will measure LDL directly. Statin adherence assessed through Vitality GlowCaps, MedSignals/Vitalsignals, Adheretech, or Wisepill (secondary outcome). The electronic pill container provides continuous data about opening and a valid approach to verifying self-administered pill taking, reflecting not only daily use but also patterns of drug use and timing. This method of adherence measurement assumes that each time the device is opened, a statin dose is taken, and that statin doses are not taken when the device is not opened. Both pill containers function just like regular pill bottles. Numerous studies have established the validity of electronic pill container measures, such as those provided by GlowCaps. Other methods have been developed to assess medication adherence, such as patient self-report, pill counts, and medication possession ratio, but there is no method that is widely recognized as superior to electronic pill containers. Although it is possible for patients to open a pill bottle but not take their statin, evidence suggests that once an individual opens a pill bottle, it is uncommon for an individual not to take the pill. However, to address this limitation of electronic pill bottles, some investigators of medication adherence have advocated for supplementing adherence data using electronic pill containers with another method of adherence assessment such as pill counts or medication refill data. In our study, we have adopted this approach of also measuring statin adherence with pharmacy refill data. Our group has extensive experience running clinical trials in which participants use electronic pill containers. We encourage participants to use the electronic pill container for study medications while continuing the usage of existing pill-organization methods such as pillboxes for other pills. Statin adherence assessed through the Medication Possession Ratio (MPR, secondary outcome) or imperfect morisky scores. Because some participants will potentially have pharmacy benefits through CVS-Caremark, we will also have data about when patients filled statin prescriptions and number of statin pills that the patients received. The MPR is calculated as the number of days for which a medication is supplied (numerator, consisting of number of statin pills provided by CVS to the participant) over a specified interval of time (denominator, for this study 365 days the duration of the interventions). The MPR will provide a secondary means to compare statin adherence across each arm. Limitations to the MPR data include 1) the problem that patients may receive the statin medication but not take the pills, which could lead to overestimation of adherence; and 2) the problem that when prescribing doctors lower the statin dose, patients may continue to use existing pills (e.g. cutting a 40mg tablet into 20 mg half-tablets and taking the half-tablets) and refill the medication at a longer interval, leading to underestimation of adherence. However, the MPR offers complementary information about adherence and will be analyzed both during the 12 months of the intervention and for an additional 6 months post-intervention. Adherence to non-statin medications assessed through the Medication Possession Ratio (secondary outcome). It is unknown whether financial incentives for statins and LDL cholesterol may lead to positive or negative spill-over effects for other medications. For instance, a focus on taking statins could lead participants to neglect the need to take their other medications. On the other hand, by helping participants reinforce good adherence habits for statins, participants might be more likely to take other medications as well. Comparisons of the MPR for non-statin medications across treatment arms will inferences about the spill-over effects of process vs. outcomes-based financial incentives. We will specifically examine spill-over effects related to other medications commonly used to lower CVD risk, e.g. blood pressure medications.

Study duration
This is a five year study. The estimated time for enrolling all subjects through completing all follow ups is 42 months. Each subject's participation is eighteen months (12 months intervention, 6 months follow-up). The proposed project dates are September 2013 - September 2018.

Resources necessary for human research protection
Describe research staff and justify that the staff are adequate in number and qualifications to conduct the research. Describe how you will ensure that all staff assisting with the research are adequately informed about the protocol and their research related duties. Please allow adequate time for the researchers to conduct and complete the research. Please confirm that there are adequate facilities for the research.
The primary study site of the proposed research project will be the Leonard Davis Institute Center for Health Incentives and Behavioral Economics (LDI CHIBE) at the University of Pennsylvania (UPENN). The LDI CHIBE is the home of the NIA-funded Penn CMU Roybal P30 Center in Behavioral Economics and Health, one of two NIH-funded Centers on behavioral economics and health in the US. These Centers provide substantial research experience, infrastructure support, and expertise in the areas of clinical trials, behavioral economics and health economics. Participants will be recruited through existing communications channels at CVS Caremark and their corporate partners. CVS has collaborated with our investigator team on several other related studies. The team includes investigators experienced in interventions to change health behaviors, clinical trials, behavioral economics, and cost-effectiveness analysis. Multiple PIs: Dr. Peter Reese is Assistant Professor of Medicine and Epidemiology at the Perelman School of Medicine (PSOM) and a member of the LDI CHIBE. He leads a portfolio of health policy research projects at the UPENN Center for Clinical Epidemiology and Biostatistics, including an RCT to improve immunosuppression adherence among kidney transplant recipients using GlowCaps bottles and the Way to Health platform. Dr. Kevin Volpp directs the LDI CHIBE and the PENN-CMU Roybal Center and is Professor of Medicine at the PSOM and Professor of Health Care Management at the Wharton School. He is a member of the National Academy of Medicine, National Academy of Sciences and has directed numerous studies of patient financial incentives to improve health behaviors. Dr. Iwan Barankay will serve as joint PI with Drs. Reese and Volpp and is Associate Professor of Management at the Wharton School and a Sloan Research Fellow. Dr. Barankay will be responsible for overseeing the creation of all data collection instruments on enrollment, oversight of study design refinement, and for overseeing data analytics in terms of both statistical analyses of effectiveness and cost effectiveness. Statistical Analysis: Dr. Mary Putt (Co-I, Statistician) is Professor of Biostatistics at UPENN and Director of two NIH-funded Biostatistics Cores at UPenn. She has 18 years of experience in the design, conduct, and analysis of clinical studies, including trials involving repeated measurements. Cost-Effectiveness Analysis: Dr. Louise Russell (Co-I) is Research Professor at the Institute for Health and Professor in the Department of Economics, Rutgers University. She is an internationally known leader in the methods of cost-effectiveness analysis. A member of the IOM since 1983, Dr. Russell co-chaired the U.S. Public Health Service Panel on Cost-Effectiveness in Health and Medicine. Analysis of LDL Reduction and Future CVD Events: Dr. Kirsten Bibbins-Domingo (Co-I) is the Lee Goldman Associate Professor of Medicine and of Epidemiology and Biostatistics at the University of California, San Francisco. She will lead analyses involving the Coronary Heart Disease Policy Model to estimate effects of trial interventions on future CVD outcomes. Consultant in Behavioral Economics: Dr. George Loewenstein is the Herbert A. Simon Professor of Economics and Psychology at Carnegie Mellon University and a founder of the fields of behavioral economics and neuroeconomics. Consultant in Health Care Communication: Dr. Punam Anand Keller is the Charles Henry Jones Third Century Professor of Marketing at the Tuck School of Business at Dartmouth. Advisory Board: Dr. Troy Brennan is Chief Medical Officer at CVS-Caremark corporation, a member of the IOM and a member of the LDI CHIBE Advisory Board; Jill Berger is Vice-President, Health & Welfare, for Marriott International and Chair of the Board of Directors for the Leapfrog Group; Thomas Pellathy is a Partner at McKinsey and Company, where he is co-leader of McKinseys efforts globally to apply behavioral economic insights to shape healthcare decision making. In-person meetings among the UPENN and Rutgers investigators will take place upon project initiation and annually thereafter. We will schedule conference calls on a quarterly basis and as needed with Carnegie Mellon and Dartmouth consultants. Ongoing project management will be facilitated by weekly meetings at UPENN and online project management software. Team members will correspond frequently via email and telephone. The UPENN investigators and staff all have offices in the same building. Team organization will resemble our approach in previous successful trials, with project leaders at the staff and faculty levels and clear lines of responsibility for achieving milestones.

Characteristics of the Study Population

Target population
Study participants will be recruited by Penn Medicine (and LGH) or CVS Caremark on behalf of the health plans as a business associate. This study will recruit participants through employer partners of CVS Caremark who meet eligibility criteria including having diabetes and/or CVD as well as statin use with medication possession ratio 80%. We used CVS claims data starting 1/1/2010 to identify individuals likely to have diabetes (on the basis of diabetes medication use) and CVD (on the basis of a previously validated algorithm that is highly specific and that uses combinations of medications commonly prescribed to patients with CVD). The CVS database reveals that out of 2,030,494 overall statin users, a total of 179,963 are classified as having diabetes and an MPR for their statin of 80%, while 70,001 are classified as having CVD and a statin MPR80%. These individuals would therefore be potentially eligible for our study. The MPR was calculated over a 365 day period.

Subjects enrolled by Penn Researchers
748

Subjects enrolled by Collaborating Researchers
0

Accrual
Potential CVS participants will be identified by reviewing CVS Caremark pharmacy claims records using our eligibility criteria of statin use and MPR80%. All potentially eligible employees within the CVS-covered group will be contacted via existing communication channels through CVS. Potentially eligible employees will have evidence of diabetes medication use and/or CVD medication use in the CVS pharmacy database. These individuals will be sent letters (paper or email) describing the study and inviting them to enroll. Participants will also be recruited from the University of Pennsylvania Health System or LGH. Potential participants will be identified by monitoring laboratory data, via weekly queries of the EPIC electronic medical record database. Primary care providers in the Penn Health System and LGH will be notified about the study and told that their eligible patients may be recruited. All eligible Penn Health System/LGH patients will be mailed letters by the study staff inviting them to sign up on the study website. Follow-up phone calls and emails will also be completed with these patients since contact information will be available through EPIC. If a participant has a direct/calculated LDL value meeting study inclusion criteria within the past 4 weeks, that value will be used as their baseline LDL and they will not need to complete the baseline lab test. Interested participants sign up for the study on line or by phone with a study coordinator based at UPENN and will be asked to provide informed consent. These individuals will have the option of entering data related to eligibility and their demographic and clinical characteristics through the Way to Health internet portal or by phone. Each potential participant will visit a local participating laboratory (either the Quest national chain of commercial laboratories or their doctor's office if they are from Penn Medicine/LGH) to confirm that their LDL is 100 mg/dl The Quest corporation has 24 hour appointment scheduling and 2,000 lab locations; these locations are in every state as well as Puerto Rico. The enrollment interview and baseline labs will allow investigators to determine final eligibility for the study. Our group has agreements in place with the Quest chain allowing research participants nationwide to have phlebotomy (blood draws). In the context of generous participation incentives (as will be provided in this study), the requirement of providing biological specimens at local laboratories has not been a barrier to program implementation. In another ongoing study, we have used Quest to enable 500 participants to get labs drawn at local laboratories. Randomization will be performed through the Way to Health platform. Randomization will be stratified by employer (should more than one employer be involved to recruit a sufficient number of participants) and study device and will use block randomization with variable block sizes. After reviewing the intake data and LDL, research staff will notify each participant of assignment using their preferred means of communication. All participants will receive an electronic pill container through the mail and will receive careful instructions about study procedures for their assigned arm. Throughout the study, coordinators will remain available to answer questions about the device use and other study procedures. Power and Sample Size: A wealth of data from clinical trials of statin medications indicates that lowering LDL cholesterol by increments of even 10 mg/dl can lead to meaningful declines in CVD risk. A meta-analysis by the Cholesterol Treatment Trialists Collaboration

suggests that a reduction in 10 mg/dl in LDL potentially reduces CVD events by 5%. We present results from a two-step approach to the power calculation intended to detect differences of 10 mg/dl or greater between the control and each intervention arm, as well as a 5 to 10 mg/dl difference between the most and least effective intervention arms. Thus Step 1 compares each of the intervention arms versus control (3 comparisons); Step 2 compares each of the intervention arms to each other arm (3 additional comparisons). For these sample size estimates, we assumed a standard deviation of 28 mg/dl (based on a prior RCT).. The nominal experiment-wise two-sided Type 1 error rate of 0.05 was maintained in step 1, by using a Bonferroni correction (i.e., alpha=0.017, derived as 0.05/3 for 3 comparisons) and in Step 2 by using Tukeys honest significant difference. Many possibilities exist for differences between the three interventions and control; the table below considers three possibilities for a sample size of 160 per group. For differences of 10 mg/dl or larger between intervention and control, simulation studies indicate at least 80% power to detect differences between all 3 interventions and control. For differences of at least 10 mg/dl between interventions, we have at least 90% power to detect at least one difference among arms; for differences of 5 mg/dl or larger we have 82% power to detect at least one difference among arms. To ensure our sample size of 160 for the 12 month endpoint, we assumed a dropout rate of 14.5 % and inflated the sample size to 187 in each arm (n=748 in the entire study) . Sensitivity Analyses: Between November 2015 and July 2016 the device failed at rates that exceeded rates obtained in our earlier studies. We anticipated that this might increase the variance of the primary outcome, with some subjects perhaps responding positively with increased compliance to statin medication and some negatively with decreased compliance. Positive responses might reflect increased intervention from study staff, or the swapping out of one device for another spurring increased compliance. Negative responses might reflect frustration, particularly on the Process arm or the Process & Outcome arm, with the inconsistent devices. We note that our completion rates for lab visits are close to our expected rates of 85%, suggesting that the device issue has not had an impact on subject retention. APPROACH 1: As a sensitivity analysis, we increased our assumed variance by 7% (SD 29 versus SD of 28) and we decreased our effect size in the Process alone and the Process & Outcome arms by 10%. Given that only XX subjects were affected for over a month, we believe this to be a very conservative calculation of the negative impact of the device issue. Table 2 above shows that, while our power has declined somewhat, we still have close to 80% power or APPROACH 2: As a sensitivity analysis, we increased our assumed variance by 15% (SD 30 versus SD of 28). Given that only XX subjects were affected for over a month, we believe this to be a reasonable assumption. Our study was designed to detect differences of 10 mg/dl between control and treated group as this difference is deemed clinically relevant. Table 3 below shows there is some decline in power, but overall we retain good power to complete the study successfully under a number of scenarios.

Key inclusion criteria

Individuals at high risk of a cardiac event, specifically one of the following: Individuals with clinical ASCVD (defined as diagnosis with myocardial infarction, stroke, or peripheral vascular disease) with an LDL greater than or equal to 100 mg/dl ; Individuals with Diabetes (between the ages of 40-75) with an LDL greater than or equal to 100 mg/dl; Individuals without clinical ASCVD or diabetes with LDLC with an LDL greater than or equal to 100 mg/dl and estimated 10-year ASCVD risk 7.5%; Individuals without clinical ASCVD or diabetes with LDL cholesterol 190 mg/dl A prescription filled for a statin medication within the last 12 months (derived from pharmacy records or EHR); Imperfect statin adherence level as defined by one of the following: Medication Possession Ratio (MPR) less than or equal to 80% A score 0 on the 8-item Morisky Medication Adherence Questionnaire completed during enrollment

Key exclusion criteria

Patients will be excluded if they 1) are under 18 years old; 2) have a contraindication to further statin use or have suffered statin side effects, such as myopathy; 3) will not or cannot give consent; 4) have a history of active or progressive liver disease or 5) are participating in another clinical trial with related aims, 6) have co-morbidities likely to lead to death within a short period (e.g. metastatic cancer), or 7) are prescribed by their doctor PCSK9 inhibitor injections coupled with statin therapy.

Vulnerable Populations

	**Children Form**
	**Pregnant women (if the study procedures may affect the condition of the pregnant woman or fetus) Form**
	**Fetuses and/or Neonates Form**
	**Prisoners Form**
	**Other**
x	**None of the above populations are included in the research study**

The following documents are currently attached to this item:

There are no documents attached for this item.

Populations vulnerable to undue influence or coercion
All participants will be mentally able, literate, working adults participating in the study of their own free will. This population is not unusually vulnerable.

Subject recruitment
Study participants will be recruited by Penn Medicine/LGH or CVS Caremark on behalf of the health plans as a business associate in its role as a PBM. Potential CVS participants will be identified by reviewing CVS Caremark pharmacy claims records using our eligibility criteria of statin use and MPR less than 80%. All potentially eligible employees within the CVS-covered group will be contacted via existing communication channels through CVS. Potentially eligible employees will have evidence of diabetes medication use and/or CVD medication use in the CVS pharmacy database. These individuals will be sent letters (paper or email) describing the study and inviting them to enroll. Participants will also be recruited from the University of Pennsylvania Health System/LGH. Potential participants will be identified by monitoring laboratory data, via weekly queries of the EPIC electronic medical record database. Primary care providers in the Penn Health System/LGH will be notified when one of their patients are eligible to participate via the secure UPHS email system. Providers will have one week to opt patients out of receiving the recruitment letter if they feel the patient is not fit to participate in research. All eligible Penn Health System/LGH patients will be mailed letters by the study staff inviting them to sign up on the study website. Follow-up phone calls and emails will also be completed with these patients since contact information will be available through EPIC. If a participant has a calculated LDL value meeting study inclusion criteria within the past 4 weeks, that value will be used as their baseline LDL and they will not need to complete the baseline lab test. Interested participants will sign up online or by phone with a study coordinator based at UPENN and will be asked to provide informed consent. These individuals will have the option of entering data related to eligibility and their demographic and clinical characteristics through the Way to Health internet portal or by phone. Each potential participant will visit a local participating laboratory (through the Quest national chain of commercial laboratories or their doctor if they are from Penn Medicine/LGH) to confirm that their LDL is 100 mg/dl. The Quest corporation has 24 hour appointment scheduling and 2,000 lab locations; these locations are in every state as well as Puerto Rico. The enrollment interview and baseline labs will allow investigators to determine final eligibility for the study. Our group has agreements in place with the Quest chain allowing research participants nationwide to have phlebotomy (blood draws). In the context of generous participation incentives (as will be provided in this study), the requirement of providing biological specimens at local laboratories has not been a barrier to program implementation. In another ongoing study, we have used Quest to enable 500 participants to get labs drawn at local laboratories. Randomization will be performed through the Way to Health platform. Randomization will be stratified by employer (should more than one employer be involved to recruit a sufficient number of participants) and will use block randomization with variable block sizes. After reviewing the intake data and LDL, research staff will notify each participant of assignment using their preferred means of communication. All participants will receive an electronic pill container through the mail and will receive careful instructions about study procedures for their assigned arm. Throughout the study, coordinators will remain available to answer questions about device use and other study procedures.

Will the recruitment plan propose to use any Penn media services (communications, marketing, etc.) for outreach via social media avenues (examples include: Facebook, Twitter, blogging, texting, etc.) or does the study team plan to directly use social media to recruit for the research?
No

The following documents are currently attached to this item:

There are no documents attached for this item.

Subject compensation*
Will subjects be financially compensated for their participation?
Yes

The following documents are currently attached to this item:

There are no documents attached for this item.

If there is subject compensation, provide the schedule for compensation per study visit or session and total amount for entire participation, either as text or separate document

Participants will receive study instructions after they are randomized that detail the administration of additional incentives. Administration of Process Incentives for Daily Adherence. The process financial incentive involves a daily sweepstakes for taking the statin medication. The sweepstakes has two components a high probability/low value reward as well as a low probability/high value reward. We will assign each participant in the Process (arm 1) and Process plus Outcome (arm 3) a two-digit number. Each day, the pill container will automatically upload the previous days data about each participants statin adherence via a wireless internet connection to the Way to Health database at UPENN. We will let each participant pick a two-digit sweepstakes number. Each day, Way to Health software will compare this number with a random two-digit number generated by Way to Health to determine eligibility for sweepstakes awards. For a participant who was adherent the previous day, if the two digit number matches exactly (probability of 1 in 100), the participant will win $50. When the two-digit number does not match, but either the first digit or second digit matches, the subject will win $5 (probability of 18 in 100). The expected value of this sweepstakes is $1.40/day such that total winnings over the 12-month trial for a participant with perfect adherence will have an average expected value of $511. An important feature of the sweepstakes is that each eligible participant will receive daily messages about the amount they won. Non-adherent participants will also receive daily feedback about whether they would have won if they had been adherent. This program incorporates key aspects of optimal design, including objective and reliable confirmation of behavior change at frequent intervals, large potential payments to reinforce the target behavior, frequent reinforcement using high probability/low value awards, and the use of anticipated regret, a powerful motivator. We set the expected value of the sweepstakes at $1.40/day based on our previous success in significantly affecting weight loss and medication adherence with this design. In addition, there is evidence that a patient with established CVD has health expenditures ranging from $4,500 to as high as $30,000 per year, suggesting that $1.40/day incentives if effective, could be cost-effective due to the potential savings in avoiding future CVD events. Administration of Outcome Incentives for LDL. Participants in the outcomes arms (arms #2 and #3) will receive payments if their LDL decreases at least 10 mg/dl in the 3 months since the last visit. Over a year, these payments will be equivalent in magnitude to those in the process arm. The 3-month payment for the Outcome arm (arm #2) will be $126, while the payment for the Process with Outcomes Incentive arm (arm #3) will be half this value, or $63. Notably, if a participant in the Outcomes arm or the Process plus Outcomes arm achieves a reduction in LDL 20 mg/dl during one quarter of the trial and that reduction persists during the next quarter, the participant would be eligible for the outcome incentive at the end of both quarters, even if no further reduction in LDL took place during the second quarter. For instance, if a patient in the Outcome arm had an LDL decrease from 150 to 130 mg/dl between baseline and month 3, and also had an LDL of 130 mg/dl at month 6, that person would receive the incentive payment at month 3 and month 6. Participants in the intervention arms will be informed if they fail to meet adherence or LDL goals, and thereby are not eligible for payments. Providing this feedback may also improve adherence by taking advantage of anticipated regret. Additionally, patients will receive $50 after enrollment, initial blood work, and setting up the Glow Caps. A subset of participants will receive $100 after enrollment to test whether higher participation incentives increase recruitment rate. Participants will receive $50 for each subsequent lab check and $75 for the 12 month LDL check, for a total of $325 if

all lab tests are completed.

Study Procedures

Suicidal Ideation and Behavior
Does this research qualify as a clinical investigation that will utilize a test article (ie- drug or biological) which may carry a potential for central nervous system (CNS) effect(s)?
No

Procedures
This 4-arm RCT will compare the relative effectiveness and cost-effectiveness of process versus outcome-based financial incentives in improving LDL cholesterol in patients who are at high risk for CVD and have been prescribed statins. Adherence in all groups will be measured using an electronic pill container as a recording device. The intervention will consist of an active phase of 12 months followed by 6 additional months of observation. Participants will be randomized evenly into one of 4 arms - process, outcomes, combination process plus outcomes, usual care (see figure for arm descriptions. All potentially eligible employees within the CVS-covered group will be contacted via existing communication channels through CVS. Potentially eligible employees will have evidence of diabetes medication use and/or CVD medication use in the CVS pharmacy database. These individuals will be sent letters (paper or email) describing the study and inviting them to enroll. Potentially eligible patients recruited from Penns Health system/LGH will be contacted by the study team via letter, email and phone calls. Interested participants will sign up online or by phone with a study coordinator based at UPENN and will be asked to provide informed consent. These individuals will have the option of entering data related to eligibility and their demographic and clinical characteristics through the Way to Health internet portal or by phone. Each potential participant will visit a local participating laboratory (through the Quest national chain of commercial laboratories or their doctor if they are from Penn Medicine/LGH) to confirm that their LDL is 100 mg/dl. The Quest corporation has 24 hour appointment scheduling and 2,000 lab locations; these locations are in every state as well as Puerto Rico. The enrollment interview and baseline labs will allow investigators to determine final eligibility for the study. Our group has agreements in place with the Quest chain allowing research participants nationwide to have phlebotomy (blood draws). In the context of generous participation incentives (as will be provided in this study), the requirement of providing biological specimens at local laboratories has not been a barrier to program implementation. In another ongoing study, we have used Quest to enable 500 participants to get labs drawn at local laboratories. Randomization will be performed through the Way to Health platform. Randomization will be stratified by employer (should more than one employer be involved to recruit a sufficient number of participants) and will use block randomization with variable block sizes. After reviewing the intake data and LDL, research staff will notify each participant of assignment using their preferred means of communication. All participants will receive an electronic pill container through the mail and will receive careful instructions about study procedures for their assigned arm. Throughout the study, coordinators will remain available to answer questions about devices use and other study procedures. LDL Measurement Schedule. Participants will have six LDL laboratory checks, at months 0, 3, 6, 9, 12 and 18 months. To maximize the likelihood that these LDL measurements are completed, the Way to Health participant tracking system will remind the study coordinators when each participant is due to visit a lab. We will obtain extensive contact information including preferred mode of communication - from each participant and update it at each follow-up visit. Participants who miss scheduled LDL checks will receive four reminders. Patients will receive $50 after enrollment, initial blood work, and setting up the device. A subset of participants will receive $100 after enrollment to test whether higher participation incentives increase recruitment rate. Participants will receive $50 for each subsequent lab check and $75 for the 12 month LDL check (the primary outcome). Administration of Process Incentives for Daily Adherence. The process financial incentive involves a daily sweepstakes for taking the statin medication. The sweepstakes has two components a high probability/low value reward as well as a low probability/high value reward. We will assign each participant in the Process (arm 1) and Process plus Outcome (arm 3) a two-digit number. Each day, the electronic pill container will automatically upload the previous days data about each participants statin adherence via a wireless internet connection to the Way to Health database at UPENN. We will let each participant pick a two-digit sweepstakes number. Each day, Way to Health software will compare this number with a random two-digit number generated by Way to

Health to determine eligibility for sweepstakes awards. For a participant who was adherent the previous day, if the two digit number matches exactly (probability of 1 in 100), the participant will win $50. When the two-digit number does not match, but either the first digit or second digit matches, the subject will win $5 (probability of 18 in 100). The expected value of this sweepstakes is $1.40/day such that total winnings over the 12-month trial for a participant with perfect adherence will have an average expected value of $511. An important feature of the sweepstakes is that each eligible participant will receive daily messages about the amount they won. Non-adherent participants will also receive daily feedback about whether they would have won if they had been adherent. This program incorporates key aspects of optimal design, including objective and reliable confirmation of behavior change at frequent intervals, large potential payments to reinforce the target behavior, frequent reinforcement using high probability/low value awards, and the use of anticipated regret, a powerful motivator. We set the expected value of the sweepstakes at $1.40/day based on our previous success in significantly affecting weight loss and medication adherence with this design. In addition, there is evidence that a patient with established CVD has health expenditures ranging from $4,500 to as high as $30,000 per year, suggesting that $1.40/day incentives if effective, could be cost-effective due to the potential savings in avoiding future CVD events. Administration of Outcome Incentives for LDL. Participants in the outcomes arms (arms #2 and #3) will receive payments if their LDL decreases at least 10 mg/dl in the 3 months since the last visit. Over a year, these payments will be equivalent in magnitude to those in the process arm. The 3-month payment for the Outcome arm (arm #2) will be $126, while the payment for the Process with Outcomes Incentive arm (arm #3) will be half this value, or $63. Notably, if a participant in the Outcomes arm or the Process plus Outcomes arm achieves a reduction in LDL 20 mg/dl during one quarter of the trial and that reduction persists during the next quarter, the participant would be eligible for the outcome incentive at the end of both quarters, even if no further reduction in LDL took place during the second quarter. For instance, if a patient in the Outcome arm had an LDL decrease from 150 to 130 mg/dl between baseline and month 3, and also had an LDL of 130 mg/dl at month 6, that person would receive the incentive payment at month 3 and month 6. Participants in the intervention arms will be informed if they fail to meet adherence or LDL goals, and thereby are not eligible for payments. Providing this feedback may also improve adherence by taking advantage of anticipated regret. Outcomes. LDL cholesterol. The primary outcome will be change in LDL cholesterol between baseline (study enrollment) and 12 months. We will also measure LDL cholesterol at 18 months three months after interventions have ended. The 18 month measurement will allow us to ascertain the durability of lipid-lowering effects attributable to financial incentives. LDL cholesterol is a strong risk factor for CVD, and reductions in LDL for instance, through statin treatment lead to meaningful reductions in the risk of cardiovascular events. LDL cholesterol is easily assessed through a blood test. LDL can be calculated indirectly from other measured lipids if the patient is fasting, or measured directly. We will measure LDL directly. Statin adherence assessed through Vitality GlowCaps, MedSignals, Adheretech, or Wisepill (secondary outcome). The electronic pill container provides continuous data about bottle opening and a valid approach to verifying self-administered pill taking, reflecting not only daily use but also patterns of drug use and timing. This method of adherence measurement assumes that each time the device is opened, a statin dose is taken, and that statin doses are not taken when the cap is not opened. The electronic pill containers function just like regular pill bottles. Numerous studies have established the validity of electronic pill container measures, such as those provided by GlowCaps. Other methods have been developed to assess medication adherence, such as patient self-report, pill counts, and medication possession ratio, but there is no method that is widely recognized as superior to electronic pill containers. Although it is possible for patients to open a pill bottle but not take their statin, evidence suggests that once an individual opens a pill bottle, it is uncommon for an individual not to take the pill. However, to address this limitation of electronic pill bottles, some investigators of medication adherence have advocated for supplementing adherence data using electronic pill containers with another method of adherence assessment such as pill counts or medication refill data. In our study, we have adopted this approach of also measuring statin adherence with pharmacy refill data . Our group has extensive experience running clinical trials in which participants use GlowCaps. We encourage participants to use the electronic pill container for study medications while continuing the usage of existing pill-organization methods such as pillboxes for other pills. Statin adherence assessed through the Medication Possession Ratio (MPR, secondary outcome). Because participants may have pharmacy benefits through CVS-Caremark, we will also have data about when patients filled statin prescriptions and number of statin pills that the patients received. The MPR is calculated as the number of days for which a medication is supplied (numerator, consisting of number of statin pills provided by CVS to the participant) over a specified interval of time (denominator, for this study 365 days the duration of the interventions). The MPR will provide a secondary means to compare statin adherence across each arm. Limitations to the MPR data include 1) the problem that patients may

receive the statin medication but not take the pills, which could lead to overestimation of adherence; and 2) the problem that when prescribing doctors lower the statin dose, patients may continue to use existing pills (e.g. cutting a 40mg tablet into 20 mg half-tablets and taking the half-tablets) and refill the medication at a longer interval, leading to underestimation of adherence. However, the MPR offers complementary information about adherence and will be analyzed both during the 12 months of the intervention and for an additional 6 months post-intervention. Adherence to non-statin medications assessed through the Medication Possession Ratio (secondary outcome). It is unknown whether financial incentives for statins and LDL cholesterol may lead to positive or negative spill-over effects for other medications. For instance, a focus on taking statins could lead participants to neglect the need to take their other medications. On the other hand, by helping participants reinforce good adherence habits for statins, participants might be more likely to take other medications as well. Comparisons of the MPR for non-statin medications across treatment arms will inferences about the spill-over effects of process vs. outcomes-based financial incentives. We will specifically examine spill-over effects related to other medications commonly used to lower CVD risk, e.g. blood pressure medications. Potential confounders and mediators. Randomization should balance measured and unmeasured confounders across study arms. However, we will measure potential confounders during study enrollment and compare their distributions across arms and perform adjustments if necessary. We will have data on participant demographics, self-reported socioeconomic status, co-morbidities, baseline MPR for statins and other medications, and baseline LDL cholesterol. Notably, these variables may also serve as either effect-modifiers (predicting the magnitude of an interventions effect across participant groups) or mediators (variables related to mechanisms whereby the intervention works) in the intervention-outcome pathway. For variables believed to be mediators, we would not make adjustments (see Data Analysis Plan, § IV.E.2.). Participant characteristics. Baseline data will be collected through the Way to Health internet portal and/or through phone interviews with study coordinators. Baseline data will be comprised of self-reported demographics (e.g., age, sex, race/ethnicity, income, education, marital status, employment, health insurance) and co-morbiditie and medication adherence questions. Participant Characteristics. At the 6, 12 and 18 month interviews, participants will complete a medication adherence questionnaire. During the 12-month interviews, we will also collect data from participants about time spent using the study device, getting labs drawn, and interacting with the Way to Health interface; these time estimates will be used for cost-effectiveness analyses.

The following documents are currently attached to this item:

There are no documents attached for this item.

Deception
Does your project use deception?
No

International Research
Are you conducting research outside of the United States?
No

Analysis Plan
Data analysis plans. We will explore and describe participant attributes across the 4 arms using graphical approaches and summary statistics (means, medians, standard deviations and the interquartile range). The primary analysis will be intent-to-treat using a linear regression analysis of the effect of treatment assignment on the outcome of change in LDL from baseline to 12 months. The model will be stratified by center and baseline will also be added as a covariate in order to improve efficiency of the analysis. Secondary analyses will adjust for measures of socioeconomic status (SES) and educational attainment (EA). We will explore whether SES and EA act as effect modifiers. We will assess model fit using standard linear regression diagnostics. In additional exploratory analyses, longitudinal models will examine the series of LDL values over time, to determine the shape of the trajectory and consider whether those trajectories differ by treatment group. All hypothesis tests will be two-sided. As a secondary analysis, we will compare the effect of the interventions vs. control among the subgroup of patients who are African-American and/or Hispanic. To maximize the power for this exploratory analysis, we will pool participants in all three incentive arms and compare to control participants. Missing data: Given low rates of drop-out in our other financial incentive studies and because this trial is low-risk, we anticipate low rates of drop-out. As with our other studies, we are diligent in proactively encouraging complete follow-up. Generous participation incentives, as well as multiple reminders

generated by the Way to Health infrastructure, will encourage participants to check their LDL at 3 month intervals. Notably, if participants change employers or benefit-plans, they will continue eligible to remain in the study. Given the rarity of serious side effects and the fact that participants have LDL levels greater than the optimal range for patients with diabetes or CVD, it is unlikely that prescribing physicians will terminate statin treatment. We will monitor completion rates closely during the study. If rates of missing data exceed 15% we will implement approaches aimed at increasing compliance with the protocol. Prior to commencing our analyses, we will compare drop-out and missing data across the 4 arms and will examine associations between participant characteristics and missing data rates. In secondary analyses, we will implement sensitivity analyses to investigate the maximum effect that missing data could have on our results and inferences. Sensitivity Analyses, Device Failure: While the primary analysis is intent to treat, we will characterize our subjects in a number of ways to explore possible impacts of the period with higher rates of device failure. Our study is not powered for hypothesis tests of these effects, and thus the analysis will be exploratory. The following analyses will be conducted: 1. We will stratify the primary analysis by whether a subject experienced two or more weeks of time in the window of time when the devices failed at rates higher than previous studies. Estimates and 95% CI of the effect sizes for the two strata will be determined with the goal of determining whether is evidence of a reduction in effect size due to an excess of faulty devices. 2. For each subject we will create a variable quantifying the proportion of their treatment time in the window with properly functioning devices. For most subjects, this variable will be 1, but for a subjects with say 2 months in the window of faulty devices, this would be 61/356=.83. We will additionally include a variable indicating whether a subject required a device swap. These variables will be included in the primary regression model. Of interest is whether the LDL levels at 12 months (change from baseline) are associated with either variable, and whether the association between LDL level at 12 months and treatment group is confounded by either variable. Additionally, we anticipate that any effect of device malfunction should primary affect the response of subjects on the Process or the Process & Outcome arms. We will thus ¬¬repeat our analyses separately with these two arms. 3. We will use a logistic regression to determine whether the likelihood of completing the study (lab visit at 12 months) is associated with the proportion of time with a properly functioning device, or the occurrence of a device swap. 4. We will explore patterns of adherence over time as a function of device type. Unfortunately having a faulty device means that we do not have reliable adherence data during the period of device failure. Thus questions will be explored using regression models with data from the periods when the devices were functioning normally. These include: a. Do mean rates of compliance differ by device for subjects randomized to a single device for the entire study period. Does the decline over time in compliance differ by device for subjects randomized to a single device over the study period. b. On average, during the period when our devices returned to normal functioning, are compliance rates similar for subjects who were exposed to faulty devices versus subjects who had normally functioning devices. These will be GEE models with a binary outcome for each day (compliant or not). The predictors include time on study, an indicator variable of whether the subject was on study during the period when the devices failed (or a quantitative variable indicating proportion of time with a functioning device), and an indicator variable for whether subjects experienced more than one device. The analysis will be carried out for the study as a whole (including a treatment indicator), and for each treatment group. Cost effectiveness analysis. Measurement of costs and cost effectiveness. For our initial analysis, we will take a payer perspective. We will complete a within-trial analysis comparing incremental costs and incremental change in LDL in each arm, compared with control, during the 12 months of the trial. As a secondary analysis, we will substitute a societal perspective, which will include costs to patients, such as transportation and the time the patient devotes to the intervention. For both these analyses, costs will include: (1) incentive payments to participants (excluding incentives specific to the research); (2) costs of lab tests for those interventions in which lab tests are necessary to decide whether the participant has earned an incentive payment; (3) operational costs of implementing the interventions, including staff time administering the incentives and the computer platform to deliver the incentives (the Way to Health platform used in this study or an alternative that might be used by an employer); and (4) costs of the adherence measurement device (the electronic pill bottle and fees for connecting to the computer platform). We will also include the drug costs associated with statin medication use when adherence improves. Following usual practice in cost-effectiveness analysis we will conduct sensitivity analyses to assess the impact of uncertainty about these data, e.g., the standard error of the estimate of effectiveness, and to explore variations that might occur across employers. For the societal perspective cost-effectiveness analysis, we will follow the recommendations of the Second Panel on Cost-Effectiveness in Health and Medicine to compute additional costs to patients related to participating in an incentives program, including medication side effects, and time spent on the intervention, including travel and waiting time (using data from American Time Use Survey); time will

be valued at appropriate wage rates.1 Long-term Effects of LDL Reductions on CVD Events and Cost-Effectiveness: The goal of lowering LDL is to prevent CVD complications. To estimate the cost and effectiveness associated with preventing cardiac outcomes, if our primary analyses of effectiveness indicate significant effects of the intervention, we will use a validated model of long-term cardiac risk, the Coronary Heart Disease (CHD) Policy Model. The CHD Policy Model is a computer-simulation, state-transition (Markov cohort) model of the incidence and prevalence of CVD (myocardial infarction, sudden death, revascularization, angina, stroke) and of the mortality and costs associated with these conditions in U.S. adults.62 The Model has been used to describe trends in CVD, project the effects of interventions to reduce CVD risk, and model the cost-effectiveness of interventions.2,3,4-5 Dr. Bibbins-Domingo, who leads the core modeling team, will adapt the Model to our study population, individuals with diabetes and/or known CVD. Based on decreases in LDL observed in the arms of this study, the Model will estimate the number and type of CVD events avoided, the quality-adjusted life years (QALYs) gained, and CVD treatment costs in each arm. We will use these estimates to calculate the cost-effectiveness of the intervention in each arm. Costs and health effects will be discounted 3% per annum. Return on investment: In addition to the cost-effectiveness analysis, if our primary analyses of effectiveness indicate significant effects of the intervention, we will conduct an analysis of potential return on investment for employers, the business case for preventing expensive health complications in the short- to medium-term. Using the CHD Policy Model we will estimate costs and savings of an incentives plan to the employer 3, 5, and 7 years after implementation. Savings will include reduced medical expenditures due to avoided CVD events and complications as well as increases in projected productivity as a result of reductions in CVD disability and death.6 Cost-effectiveness References 1. Peter J. Neumann, Gillian D. Sanders, Louise B. Russell, Joanna E. Siegel, Theodore G. Ganiats, editors, Cost-Effectiveness in Health and Medicine, second edition, New York: Oxford University Press, 2016. 2.Bibbins-Domingo K, Coxson P, Pletcher MJ, Lightwood J, Goldman L. Adolescent overweight and future adult coronary heart disease. N Engl J Med 2007;357:2371-9. 3.Odden MC, Coxson PG, Moran A, Lightwood JM, Goldman L, Bibbins-Domingo K. The impact of the aging population on coronary heart disease in the United States. Am J Med 2011;124:827-33 e5. 4. Lazar LD, Pletcher MJ, Coxson PG, Bibbins-Domingo K, Goldman L. Cost-effectiveness of statin therapy for primary prevention in a low-cost statin era. Circulation 2011;124:146-53. 5.Pletcher MJ, Lazar L, Bibbins-Domingo K, et al. Comparing impact and cost-effectiveness of primary prevention strategies for lipid-lowering. Ann Intern Med 2009;150:243-54. 6. Lightwood J, Bibbins-Domingo K, Coxson P, Wang YC, Williams L, Goldman L. Forecasting the future economic burden of current adolescent overweight: an estimate of the coronary heart disease policy model. Am J Public Health 2009;99:2230-7.

The following documents are currently attached to this item:

There are no documents attached for this item.

Data confidentiality

x **Paper-based records will be kept in a secure location and only be accessible to personnel involved in the study.**

x **Computer-based files will only be made available to personnel involved in the study through the use of access privileges and passwords.**

Prior to access to any study-related information, personnel will be required to sign statements agreeing to protect the security and confidentiality of identifiable information.

x **Wherever feasible, identifiers will be removed from study-related information.**

A Certificate of Confidentiality will be obtained, because the research could place the subject at risk of criminal or civil liability or cause damage to the subject's financial standing, employability, or liability.

A waiver of documentation of consent is being requested, because the only link between the subject and the study would be the consent document and the primary risk is a breach of confidentiality. (This is not an option for FDA-regulated research.)

x **Precautions are in place to ensure the data is secure by using passwords and encryption, because the research involves web-based surveys.**

Audio and/or video recordings will be transcribed and then destroyed to eliminate audible identification of subjects.

Subject Confidentiality
Research material will be obtained from participant interviews, the electronic pill containers adherence monitoring devices, and laboratory data from in-person blood draws for cholesterol lipid panel (including LDL). All participants will provide informed consent for access to these materials. The data to be collected include demographic data (e.g., age, sex, self-identified race), outcome data, adherence data (from the electronic pill container -if participants use the electronic pill bottle beyond the active phase of 12 months, we may analyze that adherence data), and medical conditions and medications. All measurements will be obtained according to the procedures outlined. Research material that is obtained will be used for research purposes only. The UPENN Biomedical Informatics Consortium (BMIC) will be the hub for the hardware and database infrastructure that will support the project and where the Way to Health web portal is based. The BMIC is a joint effort of the University of Pennsylvania's Abramson Cancer Center, the Cardiovascular Institute, the Department of Pathology, and the Leonard Davis Institute. The BMIC provides a secure computing environment for a large volume of highly sensitive data, including clinical, genetic, socioeconomic, and financial information. Among the IT projects currently managed by BMIC are: (1) the capture and organization of complex, longitudinal clinical data via web and clinical applications portals from cancer patients enrolled in clinical trials; (2) the integration of genetic array databases and clinical data obtained from patients with cardiovascular disease; (3) computational biology and cytometry database management and analyses; (4) economic and health policy research using Medicare claims from over 40 million Medicare beneficiaries. BMIC requires all users of data or applications on BMIC servers to complete a BMIC-hosted cybersecurity awareness course annually, which stresses federal data security policies under data use agreements with the university. Curriculum includes HIPAA training and covers secure data transfer, passwords, computer security habits and knowledge of what constitutes misuse or inappropriate use of the server. We will implement multiple, redundant protective measures to guarantee the privacy and security of the participant data. All investigators and research staff with direct access to the identifiable data will be required to undergo annual responsible conduct of research, cybersecurity, and Health Insurance Portability and Accountability Act certification in accordance with University of Pennsylvania regulations. All data for this project will be stored on the secure/firewalled servers of the BMIC Data Center, in data files that will be protected by multiple password layers. These data servers are maintained in a guarded facility behind several locked doors, with very limited physical access rights. They are also cyber-protected by extensive firewalls and multiple layers of communication encryption. Electronic access rights are carefully controlled by University of Pennsylvania system managers. We will use highly secure methods of data encryption for all transactions involving participants financial information using a level of security comparable to what is used in commercial financial transactions. We believe this multi-layer system of data security, identical to the system protecting the University of Pennsylvania Health Systems medical records, greatly minimizes the risk of loss of privacy.

Sensitive Research Information*
Does this research involve collection of sensitive information about the subjects that should be excluded from the electronic medical record?
No

Subject Privacy
Privacy refers to the person's desire to control access of others to themselves. Privacy concerns people, whereas confidentiality concerns data. Describe the strategies to protect privacy giving consideration to the following: The degree to which privacy can be expected in the proposed research and the safeguards that will be put into place to respect those boundaries. The methods used to identify and contact potential participants. The settings in which an individual will be interacting with an investigator. The privacy guidelines developed by relevant professions, professional associations and scholarly disciplines (e.g., psychiatry, genetic counseling, oral history, anthropology, psychology).
All potentially eligible employees within the CVS-covered group will be contacted via existing communication channels through CVS. Potentially eligible employees will have evidence of diabetes medication use and/or CVD medication use in the CVS pharmacy database. These individuals will be sent letters (paper or email) describing the study and inviting them to enroll. Potentially eligible patients recruited from Penns Health system/LGH will be contacted by the study team via letter, email and phone calls. Interested employees will meet by phone with a study coordinator based at UPENN and be asked to provide informed consent. These individuals will have the option of entering data related to eligibility and their demographic and clinical characteristics through the Way to Health internet portal or by phone. Each potential participant will visit a local participating laboratory (through the Quest national chain of commercial laboratories or their doctor if they are from Penn Medicine/LGH) to

confirm that their LDL is 100 mg/dl. The Quest corporation has 24 hour appointment scheduling and 2,000 lab locations; these locations are in every state as well as Puerto Rico. The enrollment interview and baseline labs will allow investigators to determine final eligibility for the study.

Data Disclosure
Will the data be disclosed to anyone who is not listed under Personnel?
Vitality, Inc., MedSignals, Adheretech, Wisepill, the companies which records the responses from the electronic pill containers. Daily adherence information will be stored on their secure computers. Quest Diagnostics, the company that will be used for blood sample collection and analysis. Lab results from Quest will be made available via a csv file that Way To Health (WTH) can download directly from secure Quest servers. The Office of Human Research Protections at the University of Pennsylvania Federal and state agencies (for example, the Department of Health and Human Services, the National Institutes of Health, and /or the Office for Human Research Protections), or other domestic or foreign government bodies if required by law and/or necessary for oversight purposes A data and safety monitoring board organized to oversee this research

*Data Protection**

x **Name**
x **Street address, city, county, precinct, zip code, and equivalent geocodes**
x **All elements of dates (except year) for dates directly related to an individual and all ages over 89**
x **Telephone and fax number**
x **Electronic mail addresses**
x **Social security numbers**
x **Medical record numbers**
x **Health plan ID numbers**
Account numbers
Certificate/license numbers
Vehicle identifiers and serial numbers, including license plate numbers
Device identifiers/serial numbers
Web addresses (URLs)
Internet IP addresses
Biometric identifiers, incl. finger and voice prints
Full face photographic images and any comparable images
Any other unique identifying number, characteristic, or code
None

Does your research request both a waiver of HIPAA authorization for collection of patient information and involve providing Protected Health Information ("PHI") that is classified as a "limited data set" (city/town/state/zip code, dates except year, ages less than 90 or aggregate report for over 90) to a recipient outside of the University of Pennsylvania covered entity?
No

Tissue Specimens Obtained as Part of Research*
Are Tissue Specimens being obtained for research?
No

Tissue Specimens - Collected during regular care*
Will tissue specimens be collected during regulator clinical care (for treatment or diagnosis)?
No

Tissue Specimens - otherwise discarded*
Would specimens otherwise be discarded?
No

Tissue Specimens - publicly available*
Will tissue specimens be publicly available?
No

Tissue Specimens - Collected as part of research protocol*
Will tissue specimens be collected as part of the research protocol?
No

Tissue Specimens - Banking of blood, tissue etc. for future use*
Does research involve banking of blood, tissue, etc. for future use?
No

Genetic testing
If genetic testing is involved, describe the nature of the tests, including if the testing is predicative or exploratory in nature. If predictive, please describe plan for disclosing results to subjects and provision of genetic counseling. Describe how subject confidentiality will be protected Note: If no genetic testing is to be obtained, write: "Not applicable."
N/A

Consent

1. Consent Process

Overview
All potentially eligible employees within the CVS-covered group will be contacted via existing communication channels through CVS. Potentially eligible employees will have evidence of diabetes medication use and/or CVD medication use in the CVS pharmacy database. These individuals will be sent letters (paper or email) describing the study and inviting them to enroll. Interested employees will meet by phone with a study coordinator based at UPENN and be asked to provide informed consent. These individuals will have the option of entering data related to eligibility and their demographic and clinical characteristics through the Way to Health internet portal or by phone.

Children and Adolescents
There will be no children or adolescents enrolled in this study.

Adult Subjects Not Competent to Give Consent
Adult subjects will be competent to give informed consent.

2. Waiver of Consent

Waiver or Alteration of Informed Consent*
Waiver of written documentation of informed consent: the research presents no more than minimal risk of harm to subjects and involves no procedures for which written consent is normally required outside of the research context

Minimal Risk*

Impact on Subject Rights and Welfare*

Waiver Essential to Research*

Additional Information to Subjects

Written Statement of Research*
Yes

If no written statement will be provided, please provide justificiation

The following documents are currently attached to this item:

There are no documents attached for this item.

Risk / Benefit

Potential Study Risks
Risks Involved in the Main Study. There are minimal risks associated with providing sweepstakes-based financial incentives to improve adherence to statins among individuals already prescribed this medication by their physicians. The main risk is loss of confidentiality, which will be protected as described below. Another small risk exists for local trauma (hematoma) during blood draw for cholesterol level /lipid testing. However, this is a minimal risk since blood will be drawn by skilled phlebotomists through Quest Diagnostics. There are no potential risks associated with any other measures or data to be collected. During the consent process, we will inform subjects of the risks associated with blood draws and loss of confidentiality. The following measures will be taken to minimize risks in this trial. First, enrollment criteria will limit participation to individuals who a) have been prescribed a statin prior to enrollment (confirmed through pharmacy records), b) deny a history of active liver disease, and c) deny allergies or adverse events to statins (including myopathy).. Third, participants will be informed about risks of liver and muscle inflammation with use of statins during the informed consent process. Fourth, during the trial, we will communicate with participants at 3-month intervals through Way to health and ask specifically about symptoms of muscle inflammation or new diagnoses of muscle or liver disease. Participants will also have the option of spontaneously reporting possible drug-associated complaints at any time. For the revised application, we have also reduced the expected value of the financial incentive from $2.80 to $1.40/day, which should reduce the possibility of undue incentive to continue the medication were side effects to develop. Lastly, we will alert prescribing physicians when any participant develops evidence of a clinically significant statin side-effect. In summary, given the low incidence of serious adverse effects with statins, our screening measures, the fact that participants have already been prescribed a statin, and the reduction in the magnitude of incentives, this intervention should confer substantial potential benefit but little incremental risk. No results will be reported in a personally identifiable manner. Electronic Data Security. The same procedure used for the analysis of automated data sources to ensure protection of patient information will be used for the survey data, in that patient identifiers will be used only for linkage purposes or to contact patients. The study identification number, and not other identifying information, will be used on all data collection instruments. All study staff will be reminded to appreciate the confidential nature of the data collected and contained in these databases.

Potential Study Benefits
Participants in this study may not receive any direct benefits. Some may benefit directly by improving their adherence to statin medications and thus lowering their risk for future heart attacks, strokes and death, improved quality of life, and reduced medical care costs. The control group is unlikely to directly benefit, but this group will continue to receive usual care. Knowledge gained from the study will assist in development of interventions in others who are not adherent to their prescribed medications. The potential public health impact of a successful intervention to improve adherence to statin medications is enormous and could reduce the number of deaths from heart attacks and strokes by tens of thousands in the United States each year. The risks of loss of confidentiality are minimal in this study. Thus, the benefits of this research to the participants studied, and to society at large, far surpass the risks.

Alternatives to Participation (optional)
To not participate in the study.

Data and Safety Monitoring
The data and safety monitoring plan will have three parts. First, the BMIC will develop and implement methods of verifying entered data and of quality control. Second, the PI will be directly responsible for

identifying and reporting all serious adverse events, protocol deviations/violations and unanticipated events to the IRBs and funding agency promptly, as appropriate. The PI will also report all adverse events, accrual rates, retention rates, and all other logistical issues to the DSMB (described below) at least biannually (and more frequently if there are serious adverse events). Third, there will be a DSMB responsible for monitoring the trial. A written research protocol will undergo formal institutional scientific and institutional review board (IRB) review at the University of Pennsylvania (UPENN) to ensure protection of the rights and welfare of human research subjects. Specifically, the multiple principal investigators (PIs) and the IRB will be responsible for ensuring risks to human subjects are minimized, risks are reasonable, subject selection is equitable, the research team has access to adequate resources to conduct the study, the informed consent process meets regulatory and ethical requirements, adequate provision is made to protect human subjects by monitoring the data collected and there are adequate provisions to protect subject privacy per HIPAA regulations and confidentiality of data. All senior/key personnel and research staff who will be involved in the design and conduct of the study must receive education in human research subject protection from a training program that is approved by a properly constituted independent Ethics Committee or Institutional Review Board. The multiple PIs will be responsible for ensuring that project faculty and staff have the equipment and training required to protect privacy and confidentiality and will monitor and document that these individuals are properly certified. If new senior/key personnel and staff become involved in the research, documentation that they have received the required education will be included in the annual progress reports. The UPENN Office of Regulatory Affairs currently requires HIPAA training upon designation as research investigator/staff and recertification in human research subject protection every three years. The UPENN IRB will serve as the IRB of record for any external ethics review boards or IRBs applicable to researchers from other institutions who may have access to human research subjects identified data. Data and Safety Monitoring Board. The DSMB will be composed of experts in clinical trials, medical economics, general internal medicine, and biostatistics, along with multiple PIs Drs. Reese and Volpp as non-voting members. We consider the proposed trial to be relatively low risk. Therefore, we have arranged for a monitoring committee that is assigned to review the study and staff training protocols, monitor the trial for safety and adverse events, and conduct a semi-annual meeting. These members will not be involved directly with the trial. The members that we propose to serve on this committee and their activities are: 1) Dr. Steven Wiviott, who is Assistant Professor of Medicine at Harvard Medical School, a cardiologist and member of the Thrombolysis in Myocardial Infarction (TIMI) clinical trials group. He has led multiple RCTs related to cardiovascular disease (CVD), including as PI of the multi-center, international PRINCIPLE-TIMI 44 trial that compared the effects of high-dose clopidogrel with prasugrel on platelet function and inflammation. He was also lead author of the PROVE IT-TIMI 22 substudy, which focused on the safety on the safety and efficacy of achieving very low LDL with intensive statin use; 2) Dr. Emilia Bagiella, who is Associate Professor of Biostatistics at Columbia University Mailman School of Public Health. Dr. Bagiella has extensive experience in the conduct, analysis and interpretation of clinical trials. Among other responsibilities, she serves as the co-principal investigator of the NIH funded TBI Clinical Trial Network. 3) Dr. Niteesh Choudhry, who is Associate Professor of Medicine at Harvard Medical School. His health policy research focuses on adherence to evidence-based treatments for CVD; he was the PI of the recently-completed MI FREEE trial that randomized post-myocardial infarction patients to reduced copayments for secondary prevention medications. Risk of loss of confidentiality will be minimized by making the Way to Health Portal, which contains the study consent and survey data, accessible only to trained study staff. Each subject will be assigned a unique identifier without identifying information, and any data that is exported from the portal for analysis purposes will be entered into an electronic database using only the unique identifier. Only trained study staff will have access to the code that links the unique identifier to the subjects identity. Electronic data will be stored on secure, password-protected firewalled servers at UPENN. The DSMB members will perform several duties. First, they will review and approve the research protocol and plans for data and safety monitoring prior to initiation of the study. Second, they will evaluate the progress of the trial. This will include assessment of data quality, participant recruitment, accrual and retention, participant risk versus benefit, performance of trial sites, and study outcomes. This assessment will be performed at meetings every 6 months during the clinical trials and more frequently if needed. Third, they will make recommendations to ensure that all of the issues above are appropriately addressed. The multiple PIs of the project will be responsible for responding to all recommendations of the DSMB and submitting DSMB reports to the respective IRBs.

The following documents are currently attached to this item:

There are no documents attached for this item.

Risk / Benefit Assessment

Poor statin medication adherence is a major public health problem with few scalable, cost-effective solutions. This study is designed to test two novel interventions that, if successful, will provide the research and public health communities with critically important information about these new methods. This approach has potentially broad generalizability in treating people at risk for coronary events and death nationally, as these types of incentives could be set up by insurers and broadly utilized. Moreover, the interventions can serve as a model for improving adherence among other medications for chronic diseases. Because of the large scientific and public health benefits of the knowledge gained from this study, the minimal risks to participants are reasonable in relation to the importance of the knowledge that reasonably may be expected to result.

General Attachments

The following documents are currently attached to this item:

Cover Letter (irbcoverletter_6.20.2019.doc)

Statistical Analysis Plan: Process versus Outcomes Trial

Table of Contents

1 Descriptive Analyses ... 1

2 Inference ... 1

2.1 Primary outcome ... 1

2.2 Secondary analyses of LDL-c based endpoints ... 2

2.3 Adherence & Association with ΔLDL at 12 months ... 2

2.4 Spillover Study (Amendment) ... 2

3 Sensitivity analysis, Device Failure & Use of Multiple Devices ... 3

1 Descriptive Analyses

1. Baseline characteristics. (Table 1) Describe the two populations (Penn Medicine & Lancaster General) and the sample, stratified by arm (include numbers (%) missing for each variable)
   a. Age
   b. Sex
   c. Race/ethnicity
   d. Income/household size/financial situation/marital status
   e. Education
   f. Health condition
   g. Patient Activation Measure (PAM)
2. LDL-c Descriptive statistics
   a. Baseline LDL-c (mean, median, IQR, SD, 95% CI on mean
   b. Repeat for LDL-c and change from baseline, Δ_{LDL}, at 3,6,9 months and 12 & 18 months in cross-sectional fashion
   c. Determine rate of missing LDL-c by arm at 3,6,9 months and 12 & 18 months

2 Inference

2.1 Primary outcome

- Linear regression model of primary outcome, Δ_{LDL}, for month 12 of the study as a function of study arm with baseline LDL-c and population included in the model (Penn Medicine & Lancaster General)
- Primary analysis uses multiple imputation (MI) to adjust for missing data
    - Describe subjects who are missing 12-month LDL-c data (within 60-day window)
    - Imputation model: includes arm assignment, and all of the baseline covariates described above.

- Hypothesis testing as described in the protocol and in previous Habit Formation Trial. Use a family-wise type I error rate of 0.05.
    i. Compare each arm to control using Holm-Bonferroni correction
    ii. Compare any arms that are significant (adjusted p<.05) to each other using Tukey's Honest Significant Difference.

2.2 Secondary analyses of LDL-c based endpoints

a. Primary endpoint Δ_{LDL} at 12 months.
    a. Complete case analysis. Use only those subjects without missing data at 12 months
    b. Repeat primary analysis
        i. without adjustment for baseline LDL-c or site:
        ii. with adjustment for baseline LDL-c only
        iii. with adjustment for site only
        iv. with additional adjustment for race, gender, income and education (basis demographic and socio-economic status)
b. Post-intervention of Δ_{LDL} at 18 months.: Repeat primary analysis
c. Across visits: Mixed effects model of Δ_{LDL}as a function of study visit and intervention arm. Include an interaction term between visit and intervention arm. Use intervention period only. Test for differences between each intervention arm and control across the visits.
d. Subgroup analyses for intervention period: Mixed effects models separately for subgroups of:
    i. Gender
    ii. Race
    iii. Income stratified at <\$50,00 $\geq$\$50,000
    iv. LDL-c at baseline: Use < 160 mg/dl versus $\geq$160 mg/dl. These cutoffs chosen in order to target individuals with poorer LDL-c and also to have sufficient sample size to make meaningful comparisons across arms.
    v. ASCVD or not
    vi. Penn Medicine versus Lancaster General

2.3 Adherence & Association with Δ_{LDL} at 12 months

a. Descriptive analyses. Describe measured adherence via pill bottle openings by arm, gender, race, income, baseline LDL-c. Describe measured adherence for the 12-month intervention period by arm and during final 30 & final 60 days of the intervention, again by arm.
a. Association between measured adherence and change in LDL-c at 12 months; association between measured adherence in each 30 day/quarterly interval preceding each lab visit.

2.4 Spillover Study

Our NIH grant pre-specified that we would examine spillover effects of financial incentives on other medications commonly used to lower CVD risk. We specified the endpoints of systolic blood pressure and hemoglobin A1c and the approach to measuring these outcomes after the trial had commenced but prior to the analysis of the data.

The following secondary endpoints from the Penn Medicine electronic medical record (EPIC) were examined:

1. HbA1c (restricted to subjects with diabetes diagnosis)
2. Systolic blood pressure (restricted to subjects with hypertension)
3. The goal was to explore evidence of any systematic change in these endpoints by study arm.

3 Sensitivity analysis, Device Failure & Use of Multiple Devices

We will characterize our subjects' experience to explore possible impacts of the period with higher rates of device failure. Our study is not powered for hypothesis tests of these effects. These analyses will be exploratory. The following analyses will be conducted:

a. Each subject will be coded 1/0 depending on whether their participation in the study involved a 'high device failure' period. For each stratum (and for each arm, we will create estimates and 95% CI of the mean LDL-C reduction by arm. We will estimate an alteration in effect size (difference in LDL-C reduction between arms) due to exposure to faulty devices.
b. We will create a variable, quantifying the proportion of the intervention period in the window with properly functioning devices. For most subjects, this variable will be 1 (always functional;), but for subjects with say 2 months in the calendar window of faulty devices, this would be 300/360=.83. We will compare the probability of completing the study (lab visit at 12 months) by whether the subject spent time in the window of a poorly functioning device.
c. Lastly, we used 3 different devices in this study. Of interest is how the response to the intervention may have differed by device. This will be modeled and explored.
