# Supplementary material for: Effect of Financial Incentives for Process, Outcomes, or Both on Cholesterol Level Change: A Randomized Clinical Trial
Source: JAMA Netw Open. 2021 Oct 4;4(10):e2121908. doi: 10.1001/jamanetworkopen.2021.21908 (PMC8491106; doi:10.1001/jamanetworkopen.2021.21908)
Supplement: Supplement 2. — eMethods 1. Outcome Ascertainment eMethods 2. Inclusion and Exclusion Criteria eFigure 1. Prerandomization Invitation, Screening, and Exclusion eMethods 3. List of Baseline Assessments eMethods 4. Smart Pill Bottles Use in the Trial eMethods 5. Methods Related to Administration of Financial Incentives eMethods 6. Methods Related to Secondary and Sensitivity Analyses eTable 1. Adherence Scores for Participants Enrolled in the Trial, by Study Group eTable 2. Baseline Characteristics of Participants Enrolled at Penn Medicine Sites Versus Lancaster General Hospital Sites eTable 3. Characteristics of Participants Who Did and Did Not Complete the 12-Month LDL-C Measurement eTable 4. Secondary Outcome of Change in LDL-C From Baseline to 18 Months (mg/dL) eFigure 2. Subgroup Analyses of the Primary Outcome of Change in LDL-C (mg/dL) From Baseline to 12 Months Using a Linear Mixed-Effects Model eTable 5. Secondary Complete Case Analysis for Outcome of Change in LDL-C (mg/dL) From Baseline to 12 Months Using the Same Model as Reported for the Primary Analysis eFigure 3. Secondary Analysis of Data From All Visits During the Intervention (3, 6, 9, and 12 Months) eTable 6. Sensitivity of Primary Results to Different Models; Change in LDL-C (mg/dL) From Baseline to 12 Months Using a Linear Model With Adjustment for Different Baseline Covariates eTable 7. Change in LDL-C (mg/dL) From 3-Month Visit to 12-Month Visit eFigure 4. Secondary Outcome of Mean Weekly Measured Statin Adherence, by Intervention Arm eTable 8. Secondary Outcome of Mean Weekly Measured Statin Adherence, by Intervention Arm eTable 9. Summary of Adverse Events eFigure 5. Spillover Analysis Flowchart of Penn Medicine Patients With Hypertension and Diabetes Plus Usual Care Data for Hemoglobin A1c and Systolic Blood Pressure, Respectively, in the Prerandomization Period (up to 6 Months Prior) and Later Period (9-15 Months Postrandomization) [file jamanetwopen-e2121908-s002.pdf]

Supplementary Online Content

Reese PP, Barankay I, Putt M, et al. Effect of financial incentives for process, outcomes, or both on cholesterol level change: a randomized clinical trial. *JAMA Netw Open*. 2021;4(10):e2121908. doi:10.1001/jamanetworkopen.2021.21908

eMethods 1. Outcome Ascertainment

eMethods 2. Inclusion and Exclusion Criteria

eFigure 1. Prerandomization Invitation, Screening, and Exclusion

eMethods 3. List of Baseline Assessments

eMethods 4. Smart Pill Bottles Use in the Trial

eMethods 5. Methods Related to Administration of Financial Incentives

eMethods 6. Methods Related to Secondary and Sensitivity Analyses

eTable 1. Adherence Scores for Participants Enrolled in the Trial, by Study Group

eTable 2. Baseline Characteristics of Participants Enrolled at Penn Medicine Sites Versus Lancaster General Hospital Sites

eTable 3. Characteristics of Participants Who Did and Did Not Complete the 12-Month LDL-C Measurement

eTable 4. Secondary Outcome of Change in LDL-C From Baseline to 18 Months (mg/dL)

eFigure 2. Subgroup Analyses of the Primary Outcome of Change in LDL-C (mg/dL) From Baseline to 12 Months Using a Linear Mixed-Effects Model

eTable 5. Secondary Complete Case Analysis for Outcome of Change in LDL-C (mg/dL) From Baseline to 12 Months Using the Same Model as Reported for the Primary Analysis

eFigure 3. Secondary Analysis of Data From All Visits During the Intervention (3, 6, 9, and 12 Months)

eTable 6. Sensitivity of Primary Results to Different Models; Change in LDL-C (mg/dL) From Baseline to 12 Months Using a Linear Model With Adjustment for Different Baseline Covariates

eTable 7. Change in LDL-C (mg/dL) From 3-Month Visit to 12-Month Visit

eFigure 4. Secondary Outcome of Mean Weekly Measured Statin Adherence, by Intervention Arm

eTable 8. Secondary Outcome of Mean Weekly Measured Statin Adherence, by Intervention Arm

eTable 9. Summary of Adverse Events

eFigure 5. Spillover Analysis Flowchart of Penn Medicine Patients With Hypertension and Diabetes Plus Usual Care Data for Hemoglobin A1c and Systolic Blood Pressure, Respectively, in the Prerandomization Period (up to 6 Months Prior) and Later Period (9-15 Months Postrandomization)

This supplementary material has been provided by the authors to give readers additional information about their work.

© 2021 Reese PP et al. *JAMA Network Open.*

eMethods 1: Outcome ascertainment

The schema displays data collection for the primary outcome of LDL, the secondary adherence outcome and the spillover analyses.

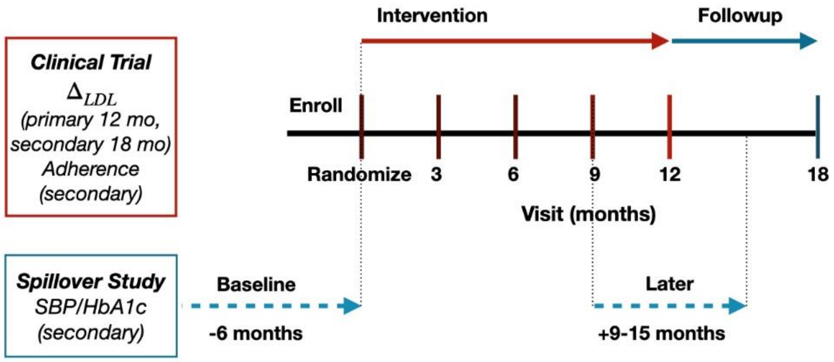

Primary outcome of LDL-c: The primary outcome was change in LDL-c (Δ_{LDL}), from baseline to a target date of 12 months, defined as 360 days (+28/-14 days). These LDL-c measurements were ordered by study personnel through affiliated labs or a national laboratory vendor, per participant preferences. Where available, study staff also collected LDL-c values measured through routine care and recorded in the electronic medical record (EMR).

An acceptable 12-month LDL outcome was defined as: an LDL measured using a study ordered lab or a lab obtained in clinical care on or after day 346 after the index date (date of randomization) and on or before 388 days after the index date. We provided a 42-day outcome measurement window in order to allow time for multiple reminders to participants to visit the lab for cholesterol measurement.

Secondary pre-specified outcome of adherence: A secondary outcome was statin adherence measured through pill bottle opening. Each day the pill bottle was opened, it electronically transmitted a signal to Way To Health. A participant could also be recorded as adherent in the database if he or she reported pill bottle malfunction and indicated that adherence data were not properly collected. In this case, staff promptly diagnosed and corrected the problem. Adherence was pre-defined as the number of daily pill bottle openings during the 12-month intervention.

Pre-specified exploratory outcome of Hemoglobin A1c (HbA1c): An exploratory outcome among patients with a diabetes diagnosis and recruited from Penn Medicine was Hemoglobin A1c. Using the electronic medical record, we collected usual care HgbA1c measurements in the baseline period (up to 6 months prior to and including the trial randomization date) and then within a 3 month window around 12 months post-randomization (i.e., between 9 and 15 months after randomization). Only individuals with at least one measurement in both periods were included. Within each period, if participants had multiple HgbA1c measurements, we used their median value. For each arm, we examined the distribution of HbgA1c in baseline period and the change from baseline to 12 months

Pre-specified exploratory outcome of systolic blood pressure: Exploratory outcomes among patients with a hypertension diagnosis and recruited from Penn Medicine were systolic and diastolic blood pressures. Only systolic blood pressure (SBP) values are reported in the manuscript. Using the electronic medical record, we collected usual care SBP measurements in the baseline period (up to 6 months prior to and including the trial randomization date) and then within a 3 month window around 12 months post-randomization (i.e., between 9 and 15 months after randomization). Only individuals with at least one measurement in both periods were included. Within each period, if participants had multiple SBP measurements, we used their median value. For each arm, we examined the distribution of SBP in baseline period and the change from baseline to 12 months

eMethods 2: Inclusion and exclusion criteria

A) <u>Eligibility criteria related to LDL cholesterol*</u>

Eligibility Criterion	Diagnosis or Risk Criterion	Population*	
		Penn Medicine	Lancaster General Health
LDL cholesterol	Any[1]	>190 mg/dl[2]	>190 mg/dl[2]
	Diabetes[1]	Initially >130; revised to >100 mg/dl[2]	>100 mg/dl[2]
	Diagnosis of clinical ASCVD[1]	Initially >130; revised to >100 mg/dl[2]	>100 mg/dl[2]
	ASCVD 10-year Risk >7.5%[1]	>100 mg/dl[2]	>100 mg/dl[2]

[1] Participants were >=18 years, except for participants with a diagnosis of diabetes who were 40 to 75 years of age
[2] Recorded as part of usual care
Abbreviations: LDL – low density lipoprotein cholesterol; ASCVD - Atherosclerotic Cardiovascular Disease

* Participants enrolled through Penn Medicine from January 2015 until March 2017. Participants enrolled from Lancaster General Health from April 2016 until March 2017.

B) <u>Eligibility related to statin use</u>

All participants attested to an active prescription to any statin medication. Patients also had to demonstrate evidence of nonadherence to the statin, which was ascertained using the 8-item Morisky Medication Adherence Questionnaire (MMAS-8). The MMAS includes 8 items that ask about medication adherence in different ways, such as asking about forgetting, skipping, and problems with adherence during travelling. Any response indicating adherence adds a point to the score. The score ranges from 0 to 8. Imperfect adherence was defined as a score less than 8 on the MMAS assessed during enrollment. A patient had to indicate with ≥1 response that they had less than perfect adherence to their statin.

1. Morisky DE, Green LW, Levine DM. Concurrent and predictive validity of a self-reported measure of medication adherence. Med Care. 1986 Jan;24(1):67-74. PMID: 3945130.
2. Morisky DE, Ang A, Krousel-Wood M, Ward HJ. Predictive validity of a medication adherence measure in an outpatient setting. J Clin Hypertens. 2008 May;10(5):348-54. PMCID: PMC2562622.

© 2021 Reese PP et al. *JAMA Network Open.*

eFigure 1. Prerandomization Invitation, Screening, and Exclusion

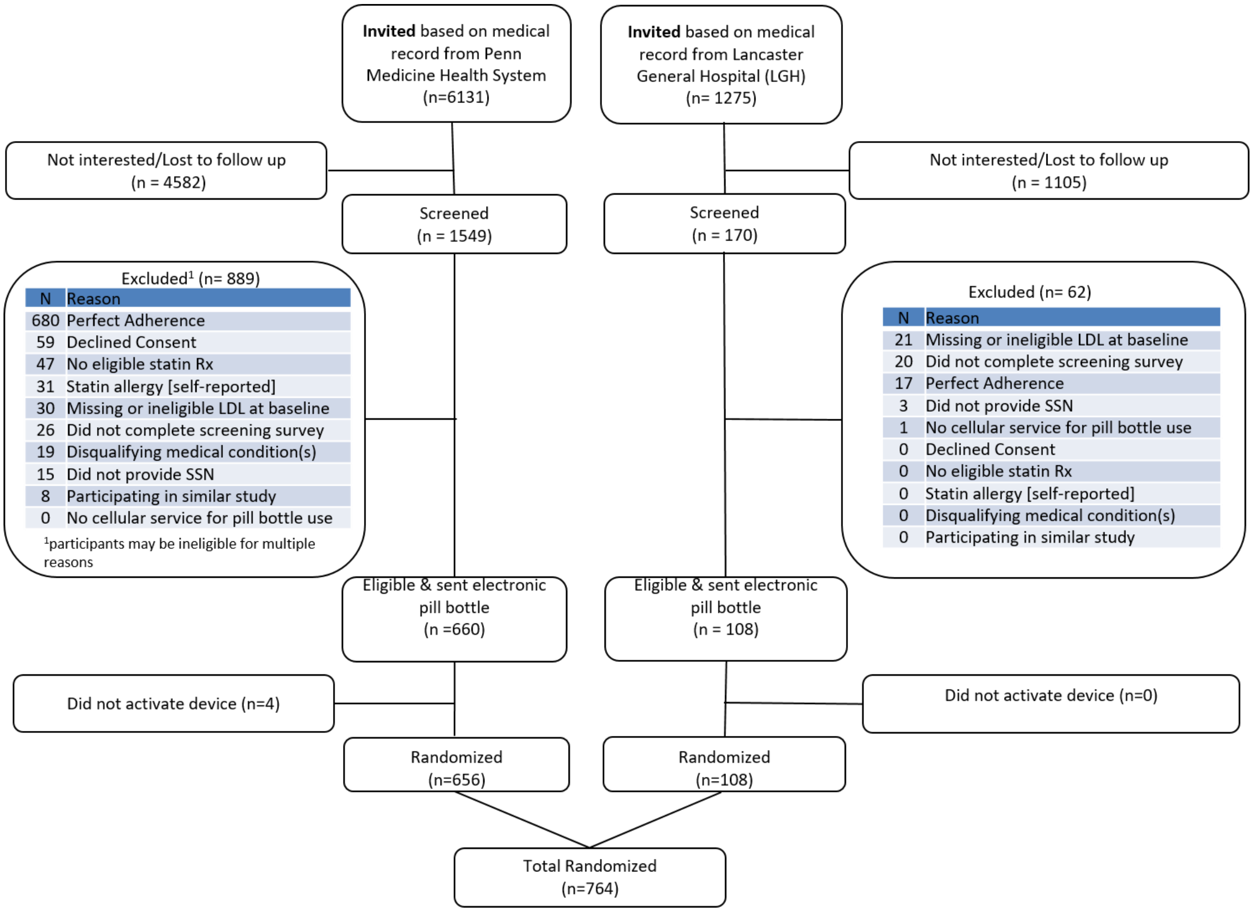

© 2021 Reese PP et al. *JAMA Network Open.*

eMethods 3: List of baseline assessments

Measure	*Description*	*Timepoint(s) of Administration*
Exclusion Questionnaire	9-item survey confirming eligibility	Baseline
Demographic Questionnaire	5-item survey	Baseline
Patience	1 question on self-rated patience	Baseline
Life Satisfaction	1 question on self-rated life satisfaction	Baseline
Statistical Questionnaire (SNS)[1]	8-item survey measuring comfort with fractions, percentages, and mathematical scenarios	Baseline
Risk	1 question on self-rated risk-tasking	Baseline/12 month
Stages of Change[2]	1 question which measures health behavioral change tailored to adherence	Baseline/6/12/18 month
Financial Decision-Making (Slack)[3]	6-item survey measuring perception of disposable income	Baseline/6/12/18 month
Incentive Questionnaire	2-item survey measuring perception of the effects of financial incentives on adherence	Baseline/6/12/18 month

1. Fagerlin, A., Zikmund-Fisher, B.J., Ubel, P.A., Jankovic, A., Derry, H.A., & Smith, D.M. Measuring numeracy without a math test: Development of the Subjective Numeracy Scale (SNS). Medical Decision Making, 2007: 27: 672-680.
2. Nigg, C.R., Burbank, P., Padula, C., Dufresne, R., Rossi, J. S., Velicer, W. F., Laforge, R. G. & Prochaska, J. O. (1999). “Stages of change across ten health risk behaviors for older adults.” The Gerontologist, 39, 473-482
3. Zauberman, G. & Lynch, J.G. (2005) Resource Slack and Propensity to Discount Delayed Investments of Time Versus Money. Journal of Experimental Psychology: General, 134, 23-37.

© 2021 Reese PP et al. *JAMA Network Open.*

eMethods 4: Smart pill bottles used in the trial

Prior to randomization, every trial participant received a smart bill bottle, which used a cellular network to transmit a record of the bottle opening to the Way to Health platform. Over the course of the trial, we sequentially provided three different bottles from different manufacturers to participants. Model 1 was removed from service with the phasing out of a cellphone network standard; the bottle was incompatible with the new standard. Model 2 was initially chosen to replace Model 1, but the manufacturer was unable to supply a sufficient quantity of devices in a timely fashion and feedback about the devices indicated some dissatisfaction among participants. The majority of our study was conducted using Model 3. A total of 589 (77%) participants used only Model 3. Including the additional 117 participants who were ultimately switched to Model 3, a total of 696 (91%) participants used Model 3 during most if not all of the study.

Pill Bottle Model	Started: N (% of Total)	Switched: N (% of Subjects Started)
1	123 (16.1%)	83 (67.5%)[a]
2	52 (6.8%)	49 (94.2%)[b]
3	589 (77.1%)	0 (0.0%)
**Total**	764 (100.0%)	132 (17.2%)

[a] 15 switched to Model 2 only; 59 switched to Model 3 only; 9 switched to model 2 and subsequently to model 3
[b] all 49 switched to Model 3

© 2021 Reese PP et al. *JAMA Network Open.*

eMethods 5: Methods related to administration of financial incentives

Over a 12-month period, individuals in the three intervention arms were eligible for financial incentives based on their daily statin adherence (Process arm), LDL-C value every 3 months (Outcomes arm), or both (Process plus Outcomes arm). We delivered statin adherence incentives as a sweepstakes to invoke people's tendency to overestimate small probabilities.[30,31] One daily bottle opening made the Process and Process plus Outcomes participants eligible for that day's incentive. For the Process and Process plus Outcomes arms, each participant was assigned a 2-digit number. Every day, a random 2-digit number was generated by the Way To Health platform and compared to eligible participants' assigned numbers to determine the size of the financial incentives. Participants in these arms were informed about their earnings the following day.[29] For the Process arm, and the Process plus Outcomes arm, we invoked loss aversion through messages to non-adherent participants about the reward that they might have missed. Incentives for LDL-c reduction in the Outcomes and Process plus Outcomes arms were delivered without a sweepstakes.

Process Arm: If both digits matched (1 in 100 probability), an adherent participant received $50. If 1 digit matched (18 in 100 probability), an adherent participant received $5. A fully adherent participant in a financial incentives arm could earn on average $1.40/day.

Outcomes Arm: Participants in the Outcomes arms received $126 each time their LDL had decreased at least 10 mg/dl in the 3 months since the last visit. Over a year, these payments were equivalent to the expected value of the process arm. A participant who achieved an LDL-c reduction $\geq$20 mg/dl during one quarter and maintained that reduction the next quarter would receive the outcome incentive at the end of both quarters, even if no further reduction in LDL-c took place during the second quarter.

Process plus Outcomes Arm: Participants were eligible for daily sweepstake rewards, but with half the expected monetary value of the Process Arm, and for 3-monthly LDL-c rewards at half the value of the Outcomes arm. The payment for the Process with Outcomes Incentive arm were half this value, or $63 and the sweepstakes winnings were $2.50 for a one-digit match and $25 for a two-digit match.

Notably, participants in all three intervention arms had the opportunity to earn $504 if they met all adherence and/or LDL reduction targets for their assigned arm

eMethods 6: Methods related to secondary and sensitivity analyses

The linear model for the primary outcome was refit using the same covariates and outcome of Δ_{LDL} from baseline to 18 months, 6 -months after the conclusion of the interventions, to assess durability of response.

We assessed differences in mean Δ_{LDL} from baseline across the four visits of the intervention period using a mixed effects model. We contrasted the control arm with each of the intervention arms using a linear combination of the 3, 6, 9 and 12-month visits. We repeated these analyses by pre-specified subgroups; no test of an interaction was used as the sample lacked statistical power to detect meaningful differences.

Using a simple linear model, we reported measured adherence for both the 12-month intervention period, and differences between any intervention arms and control.

Sensitivity analyses included (1) a complete case analysis and (2) a model adjusted for gender, education, income, and race.

As a post-hoc analysis, we assessed differences in mean Δ_{LDL} from 3-months to 6, 9, and 12-months. Models were adjusted for LDL-c at baseline and stratified by site, using multiply imputed data.

© 2021 Reese PP et al. *JAMA Network Open.*

eTable 1: Adherence scores for participants enrolled in the trial, by study group

Variable	Total (n=764)	Control (n=190)	Process (n=194)	Outcomes (n=187)	Process and Outcomes (n=187)
**Morisky Medication Adherence Questionnaire Level[a], n (%)**					
**Medium Adherence**	385 (50.4)	97 (51.1)	106 (54.6)	94 (50.3)	88 (45.6)
**Low Adherence**	379 (49.6)	93 (48.9)	88 (45.4)	93 (49.7)	105 (54.4)
**Morisky Medication Adherence Questionnaire Score, mean (SD)**	5.7 (1.4)	5.6 (1.4)	5.7 (1.4)	5.8 (1.2)	5.6 (1.4)

[a] Score 6-7 is medium adherence; < 6 is low adherence.

© 2021 Reese PP et al. *JAMA Network Open.*

eTable 2: Baseline characteristics of participants enrolled at Penn Medicine sites versus Lancaster General Hospital sites

Characteristic	Total (n=764)	Penn Medicine (n=656)	LGH (n=108)	P-value[a]
Age, mean (SD)	62.4 (10.0)	62.0 (10.0)	65.1 (9.5)	0.003
Sex [b], n (%)				0.011
Female	390 (51.2)	347 (53.1)	43 (39.8)	
Male	372 (48.8)	307 (46.9)	65 (60.2)	
Race [b], n (%)				<.001
White	440 (58.0)	341 (52.4)	99 (91.7)	
Black	271 (35.7)	268 (41.2)	3 (2.8)	
Other	48 (6.3)	42 (6.5)	6 (5.6)	
Ethnicity [b], n (%)				0.836
Hispanic or Latino	19 (2.5)	16 (2.5)	3 (2.8)	
Not Hispanic or Latino	737 (97.5)	633 (97.5)	104 (97.2)	
Education [b], n (%)				<.001
High school or less	224 (29.4)	174 (26.6)	50 (46.3)	
Some college	208 (27.3)	185 (28.3)	23 (21.3)	
College degree	330 (43.3)	295 (45.1)	35 (32.4)	
Income before taxes, n (%)				<.001
Below $50,000	303 (39.7)	293 (44.7)	10 (9.3)	
Above $50,000	338 (44.2)	329 (50.2)	9 (8.3)	
Do not wish to answer [c]	123 (16.1)	34 (5.2)	89 (82.4)	
Marital Status [b], n (%)				0.016
Single	184 (24.1)	167 (25.5)	17 (15.7)	
Married/Unmarried partners	413 (54.2)	341 (52.1)	72 (66.7)	
Divorced/Widowed	165 (21.7)	146 (22.3)	19 (17.6)	
Smoke more than 5 cigarettes per day, n (%)	62 (8.1)	55 (8.4)	7 (6.5)	0.502
Household size[b], mean (SD)	2.3 (1.2)	2.3 (1.2)	2.3 (1.1)	0.842
Diagnosed with Diabetes, n (%)	310 (40.6)	289 (44.1)	21 (19.4)	<.001
Diagnosed with ASCVD, n (%)	298 (39.0)	252 (38.4)	46 (42.6)	0.410
LDL-c Calculated, mean (SD)	138.8 (37.6)	140.4 (38.2)	128.8 (32.4)	0.003

Values are number of participants (%) or means and standard deviations (SD).
[a] Analysis of Variance (ANOVA) for continuous data and Pearson Chi-square for categorical data
[b] Values may not sum to the total number of subjects due to missing data
[c] Subjects were asked to give their annual pre-tax household income in $10,000 intervals. Those who did not wish to answer the question were asked if they were willing to share whether their income was greater or less than $50,000.

© 2021 Reese PP et al. *JAMA Network Open.*

eTable 3: Characteristics of participants who did and did not complete the 12-month LDL-c measurement

Characteristic	Total (n=764)	Completed 12-month lab (n=644)	Missed 12-month lab (n=120)	P-value of difference completed vs missed
Age, mean (SD)	62.4 (10.0)	63.2 (9.5)	58.2 (11.4)	<.01
Study group, n (%)				.11
Control	190 (24.9)	159 (24.7)	31 (25.8)	
Process	194 (25.4)	174 (27.0)	20 (16.7)	
Outcome	187 (24.5)	152 (23.6)	35 (29.2)	
P & O	193 (25.3)	159 (24.7)	34 (28.3)	
Site, n (%)				.58
Penn Medicine	656 (85.9)	551 (85.6)	105 (87.5)	
Lancaster General	108 (14.1)	93 (14.4)	15 (12.5)	
Gender, n (%)				.20
Female	390 (51.2)	335 (52.2)	55 (45.8)	
Male	372 (48.8)	307 (47.8)	65 (54.2)	
Missing	2 (0.3)	2 (0.3)	0 (0.0)	
Race, n (%)				.12
White	440 (58.0)	378 (59.2)	62 (51.7)	
Black	271 (35.7)	225 (35.2)	46 (38.3)	
Other	48 (6.3)	36 (5.6)	12 (10.0)	
Missing	5 (0.7)	5 (0.8)	0 (0.0)	
Education, n (%)				.49
High school or less	224 (29.4)	190 (29.6)	34 (28.3)	
Some college	208 (27.3)	170 (26.5)	38 (31.7)	
College degree	330 (43.3)	282 (43.9)	48 (40.0)	
Missing	2 (0.3)	2 (0.3)	0 (0.0)	
Income, n (%)				.20

© 2021 Reese PP et al. *JAMA Network Open.*

Below $50,000	694 (91.1)	581 (90.5)	113 (94.2)	
Above $50,000	68 (8.9)	61 (9.5)	7 (5.8)	
Missing	2 (0.3)	2 (0.3)	0 (0.0)	
Marital Status, n (%)				.15
Single	184 (24.1)	147 (22.9)	37 (30.8)	
Married/Unmarried partners	413 (54.2)	356 (55.5)	57 (47.5)	
Divorced/Widowed	165 (21.7)	139 (21.7)	26 (21.7)	
Missing	2 (0.3)	2 (0.3)	0 (0.0)	
Household size, mean (SD)	2.3 (1.2)	2.3 (1.2)	2.5 (1.3)	.12
Missing	6 (0.8)	6 (0.9)	0 (0.0)	
Statin adherence score, mean (SD)	5.7 (1.4)	5.7 (1.4)	5.5 (1.3)	.18
Baseline LDL-c mg/dl, mean (SD)	138.8 (37.6)	137.4 (37.9)	146.1 (35.4)	.02

© 2021 Reese PP et al. *JAMA Network Open.*

eTable 4: Secondary outcome of change in LDL-c from baseline to 18 months (mg/dL)[a]

Visit (months)	Outcome	Control arm	Process arm	Outcomes arm	Process plus Outcomes arm
18	Δ_{LDL} [b]	-34.9 (-40.0, -29.9)	-39.6 (-44.9, -34.3)	-35.9 (-41.3, -30.5)	-38.5 (-43.8, -33.3)
	Difference in change versus control [b]		-4.7 (-11.9, 2.6)	-0.9 (-8.2, 6.3)	-3.6 (-10.4, 3.2)
	P-value [c]		0.206	0.799	0.299

[a] Secondary outcome. The 18-month timepoint was 6 months post-intervention.

[b] Mean (95% CI) change from baseline from a linear model adjusted for baseline LDL-c and site. Results are reported for Penn Medicine at mean baseline LDL-c. Mean LDL-c at 18 months for LGH participants was 5.9 mg/dL higher than Penn Medicine participants. Multiple imputation was used to adjust for incomplete data.

[c] No adjustment for multiple comparisons.

© 2021 Reese PP et al. *JAMA Network Open.*

eFigure 2A-F: Subgroup analyses of the primary outcome of change in LDL-C (mg/dL) from baseline to 12 months using a linear mixed-effects model

Methods: Mean change in LDL-c was estimated by a linear mixed effects model within each subgroup using arm, visit, interaction of arm and visit, and baseline LDL-c as covariates with a random intercept term. We tested the null hypothesis that the linear combination of LDL-c change from baseline across the 3, 6, 9 and 12-month visits was identical for the control and each intervention group. Complete data were used in the analysis. Results are considered exploratory and no adjustment for multiple comparisons was made.

Forest Plots: Squares are the mean reduction in LDL-c from baseline at each timepoint, and after adjustment for the mean baseline value across all arms. Error bars are 95% confidence intervals (CI). Negative values indicate reduction in LDL-c versus baseline. Dashed line is the overall mean for the LDL-c reduction for control in the subgroup.

© 2021 Reese PP et al. *JAMA Network Open.*

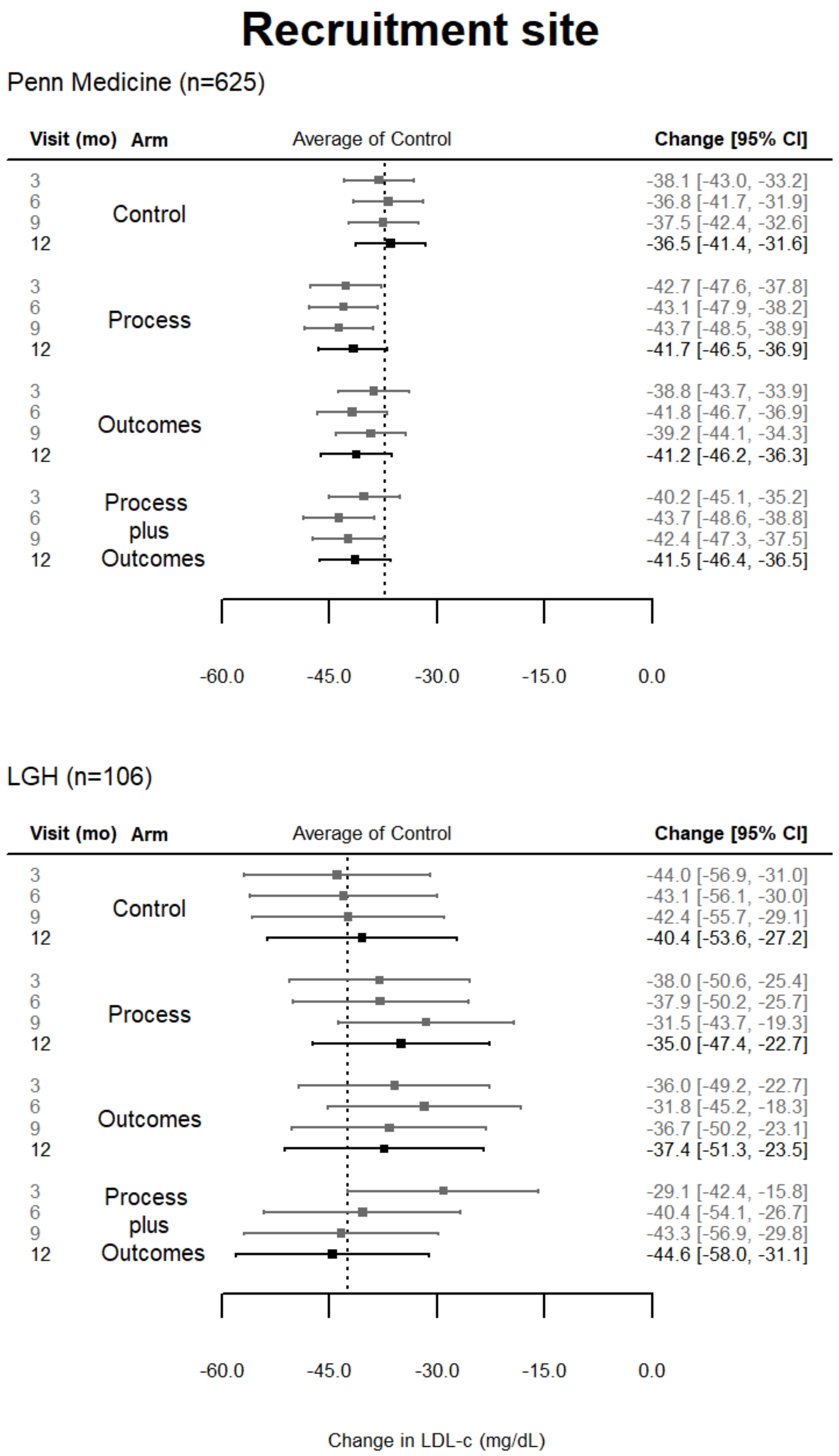

eFigure 2A: Recruitment site. For participants recruited at Penn Medicine (n=625), the results of the hypothesis test of each intervention arm versus control is Process (p=.047), Outcomes (p=.202), Process plus Outcomes (p=.063). For participants recruited at LGH (n=106) the results of the hypothesis test of each intervention arm versus control is Process (p=.362), Outcomes (p=.415), Process plus Outcomes (p=.919).

© 2021 Reese PP et al. *JAMA Network Open.*

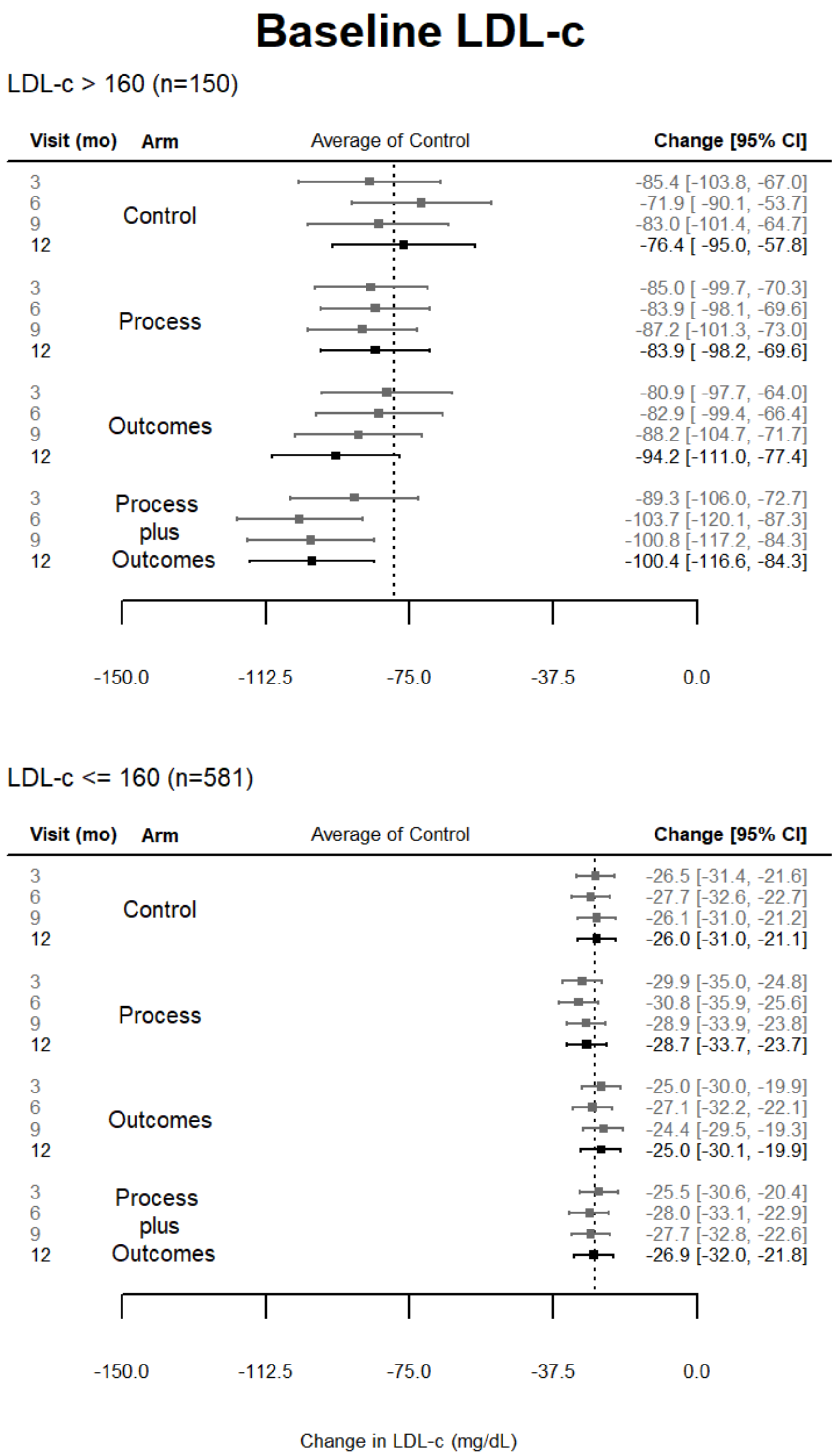

eFigure 2B: Baseline LDL-c. For those with LDL-c>160 (n=150), the results of the hypothesis test of each intervention arm versus control is Process (p=.441), Outcomes (p=.297), Process plus Outcomes (p=.002). For those with LDL-c ≤160 (n=581) the results of the hypothesis test of each intervention arm versus control is Process (p=.379), Outcomes (p=.729), Process plus Outcomes (p=.772).

© 2021 Reese PP et al. *JAMA Network Open.*

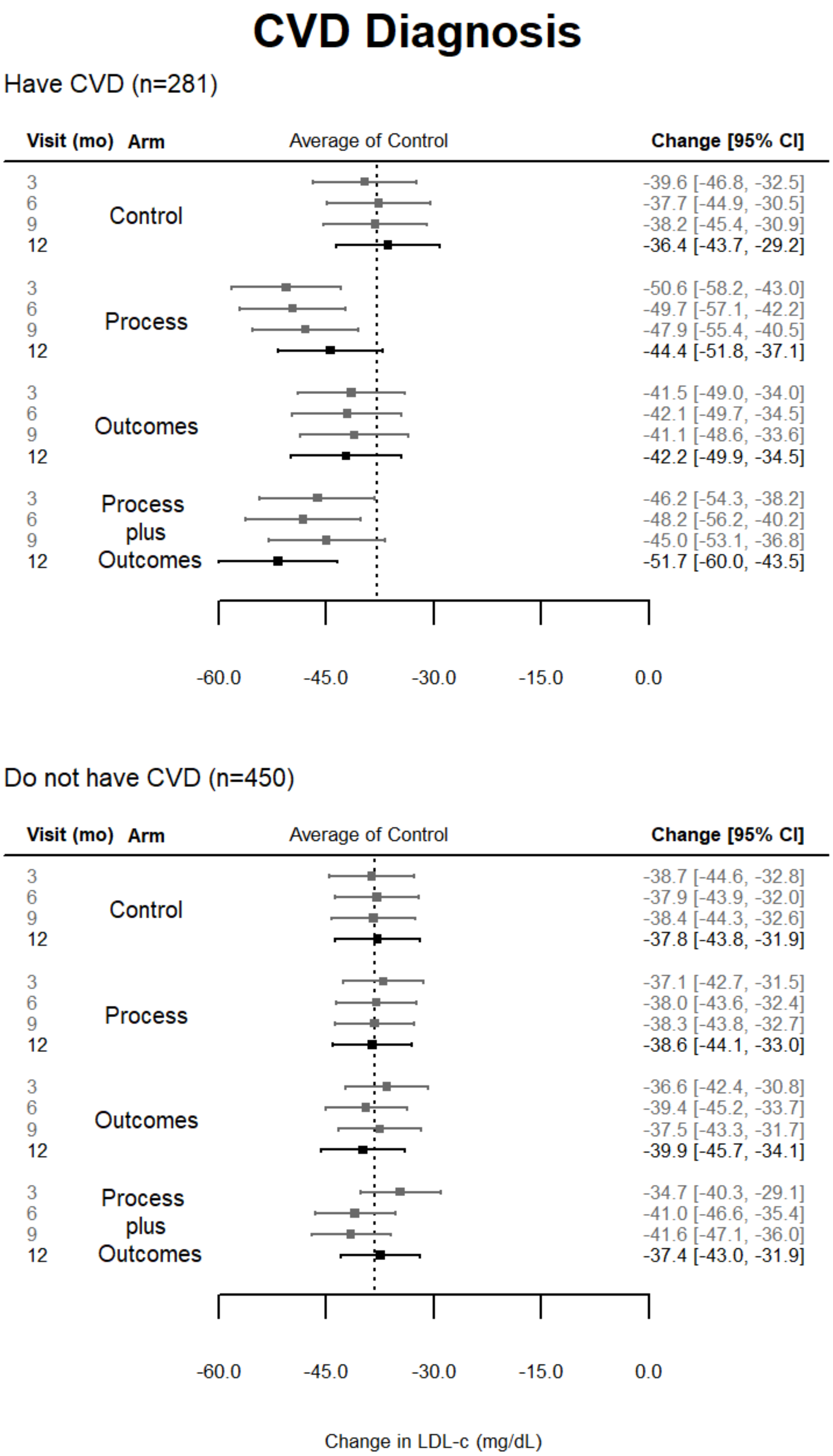

eFigure 2C: CVD diagnosis. For those with CVD (n=281) the results of the hypothesis test of each intervention arm versus control is Process (p=.026), Outcomes (p=.330), Process plus Outcomes (p=.020). For those without CVD (n=450) the results of the hypothesis test of each intervention arm versus control is Process (p=.957), Outcomes (p=.804), Process plus Outcomes (p=.583).

© 2021 Reese PP et al. *JAMA Network Open.*

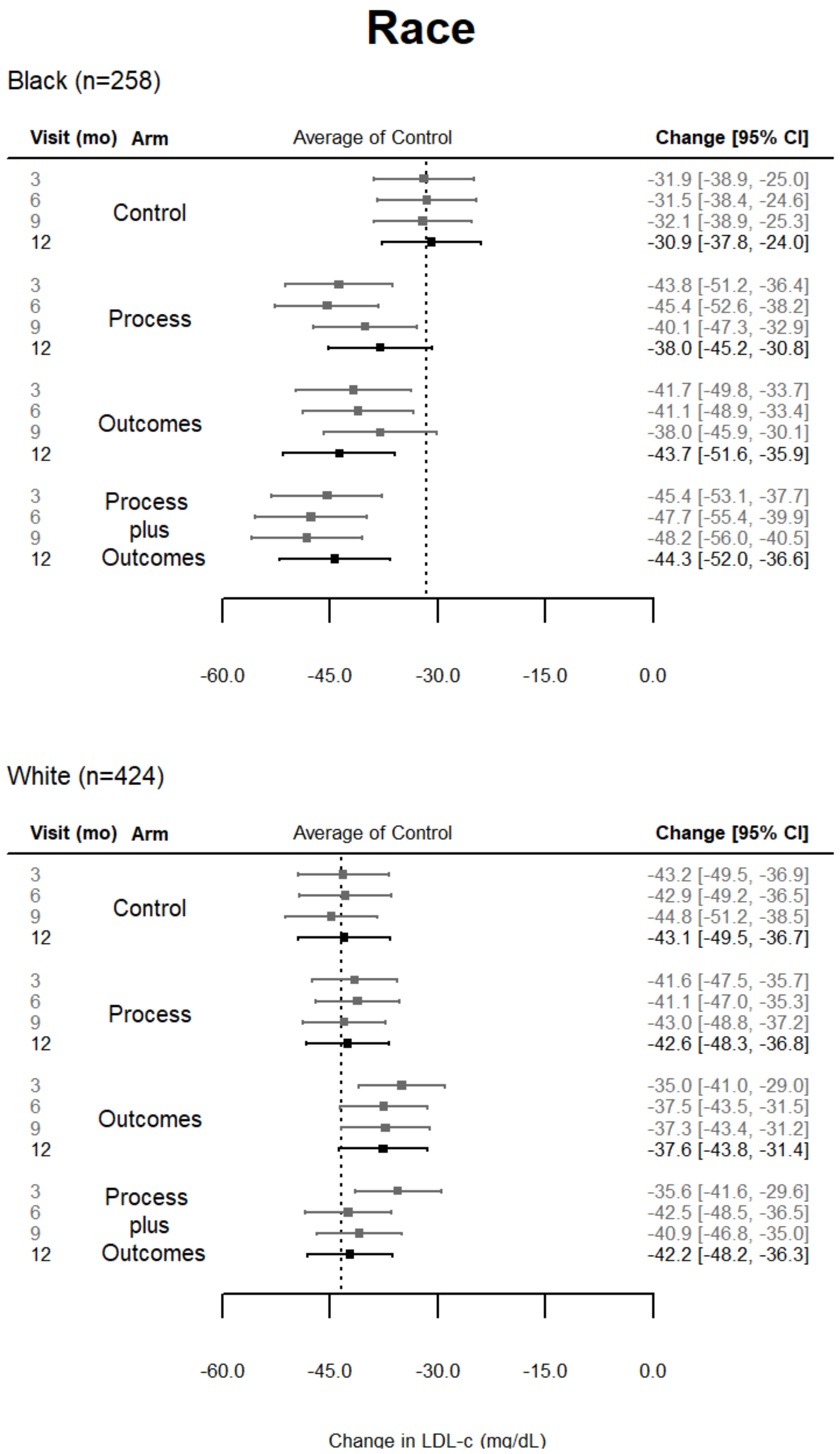

eFigure 2D: Race: Due to small sample size, participants of race category 'Other' or 'do not wish to disclose' (n=49) were excluded from this analysis. For those of Black race (n=258), the results of the hypothesis test of each intervention arm versus control is Process (p=.023), Outcomes (p=.033), Process plus Outcomes (p=.001). For those of White race (n=424) the results of the hypothesis test of each intervention arm versus control is Process (p=.720), Outcomes (p=.111), Process plus Outcomes (p=.650).

© 2021 Reese PP et al. *JAMA Network Open.*

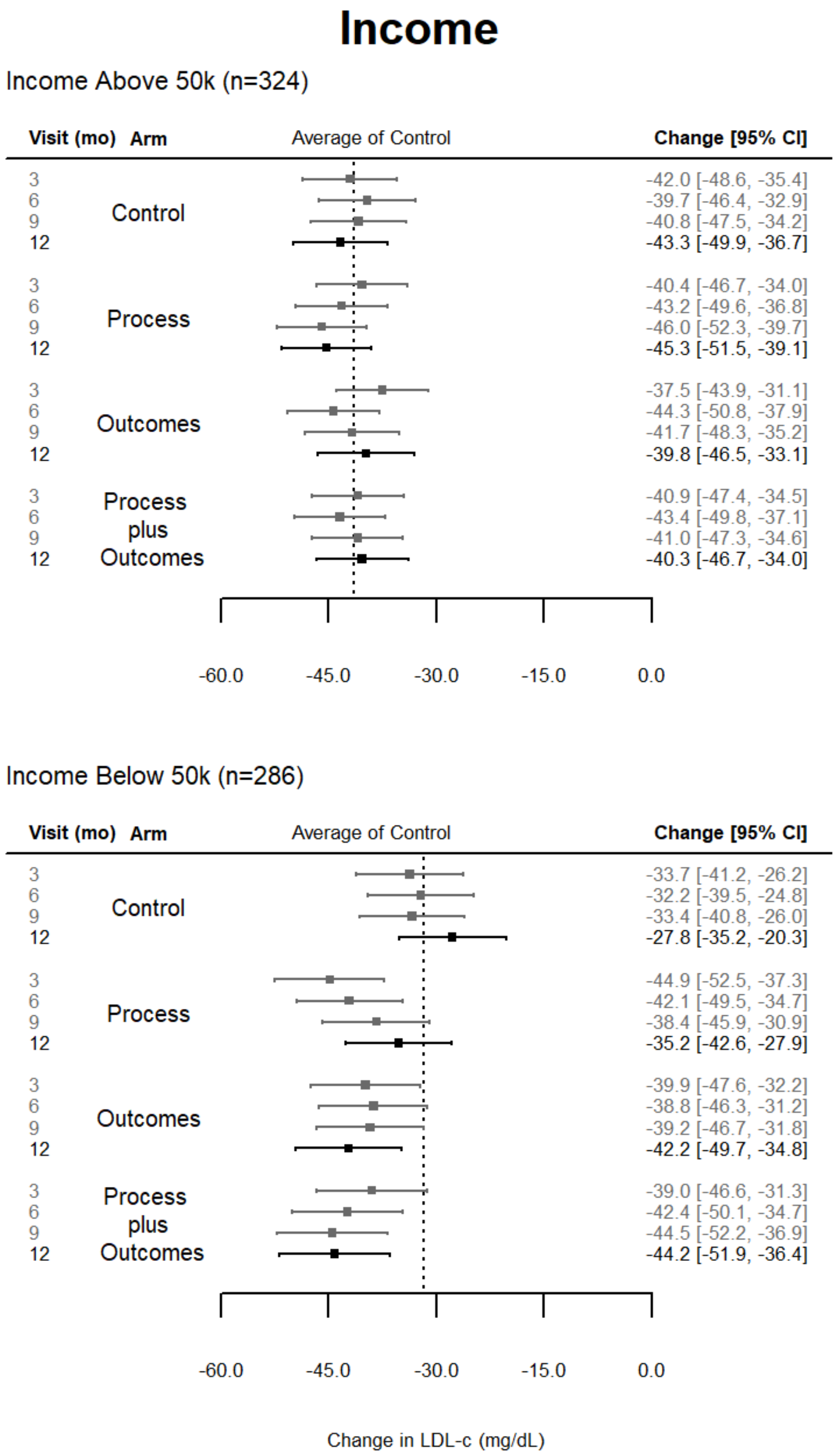

eFigure 2E: Income. Income category based on a baseline survey question asking pre-tax household income levels in $10,000 intervals with a follow-up question for subjects who did not wish to answer of: "Are you willing to share if your income is above or below $50,000?". The 121 participants who did not wish to disclose their income in response to either question are excluded from this analysis. For those with income>US $50,000 (n=324), the results of the hypothesis test of each intervention arm versus control is Process (p=.367), Outcomes (p=.869), Process plus Outcomes (p=.941). For those of income <US $50,000 (n=286) the results of the hypothesis test of each intervention arm versus control is Process (p=.097), Outcomes (p=.047), Process plus Outcomes (p=.006).

© 2021 Reese PP et al. *JAMA Network Open.*

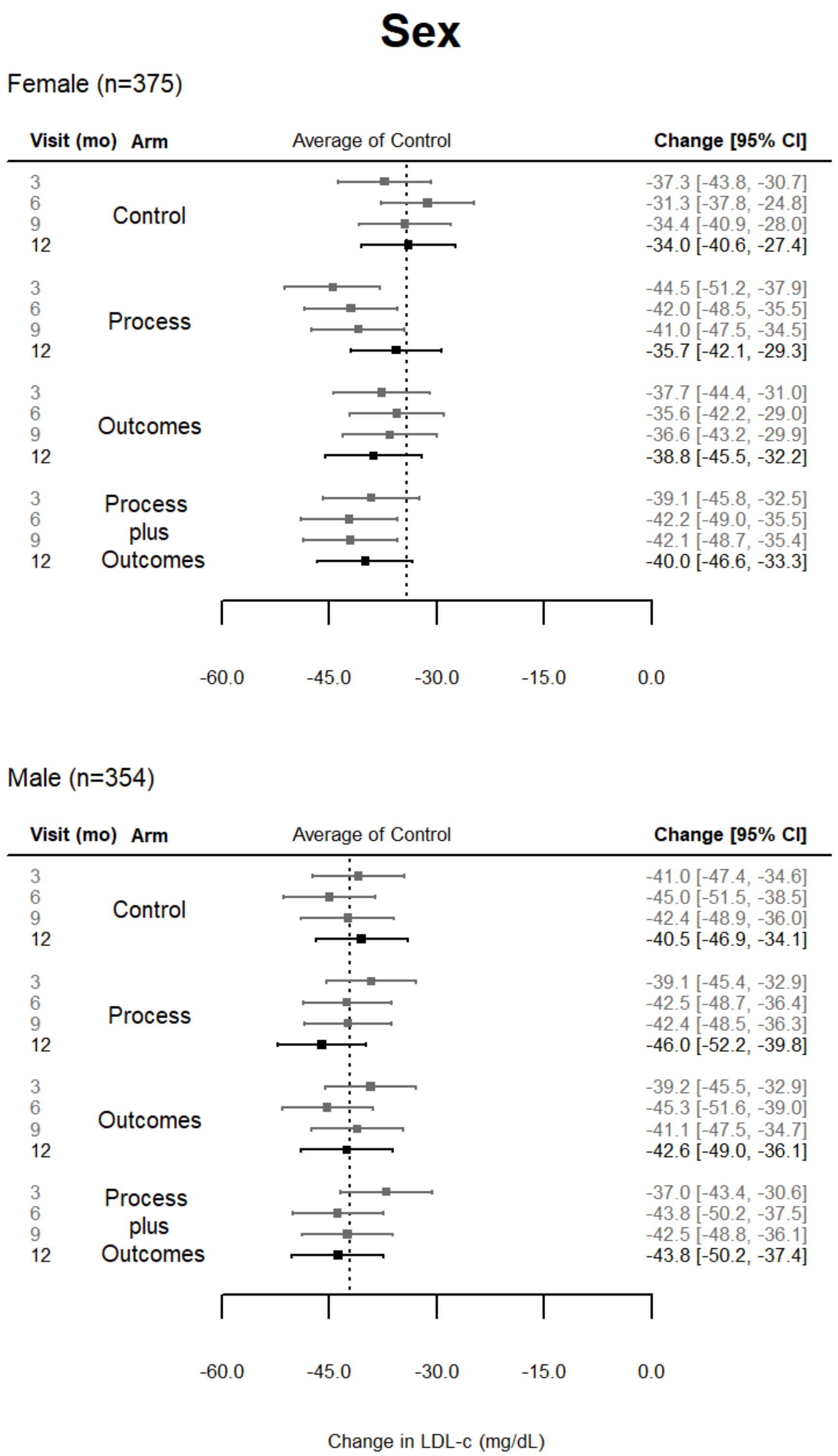

eFigure 2F: Sex. The 2 participants who did not wish to disclose their sex are excluded from this analysis. For females (n=375), the results of the hypothesis test of each intervention arm versus control is Process (p=.115), Outcomes (p=.351), Process plus Outcomes (p=.043). For males (n=354) the results of the hypothesis test of each intervention arm versus control is Process (p=.794), Outcomes (p=.930), Process plus Outcomes (p=.858).

© 2021 Reese PP et al. *JAMA Network Open.*

eTable 5: Secondary complete case analysis for outcome of change in LDL-c (mg/dL) from baseline to 12 months using the same model as reported for the primary analysis[a]

Visit (months)	Outcome	Control arm	Process arm	Outcomes arm	Process plus Outcomes arm
12	Δ_{LDL}	-36.9 (-41.7, -32.1)	-40.0 (-44.6, -35.5)	-41.5 (-46.4, -36.7)	-42.9 (-47.7, -38.1)
	Difference in change versus control		-3.1 (-9.6, 3.3)	-4.6 (-11.3, 2.1)	-6.0 (-12.6, 0.6)
	P-value		0.342	0.175	0.075

[a] Mean (95% CI) change from baseline from a linear model adjusted for baseline LDL-c and site. Results are reported for Penn Medicine at mean baseline LDL-c. Mean LDL-c at 12 months for LGH participants was 0.4 mg/dL lower than Penn Medicine participants.

© 2021 Reese PP et al. *JAMA Network Open.*

eFigure 3: Secondary analysis of data from all visits during the intervention (3, 6, 9 and 12 months)

Methods: Mean change in LDL-c was estimated by a linear mixed effects model using arm, visit, interaction of arm and visit, and baseline LDL-c as covariates, and a random intercept term. We tested the null hypothesis that the linear combination of LDL-c change from baseline across the 3, 6, 9 and 12 month visits was identical for the control and each intervention group. Complete data were used in the analysis (n=731). No adjustment for multiple comparisons was made.

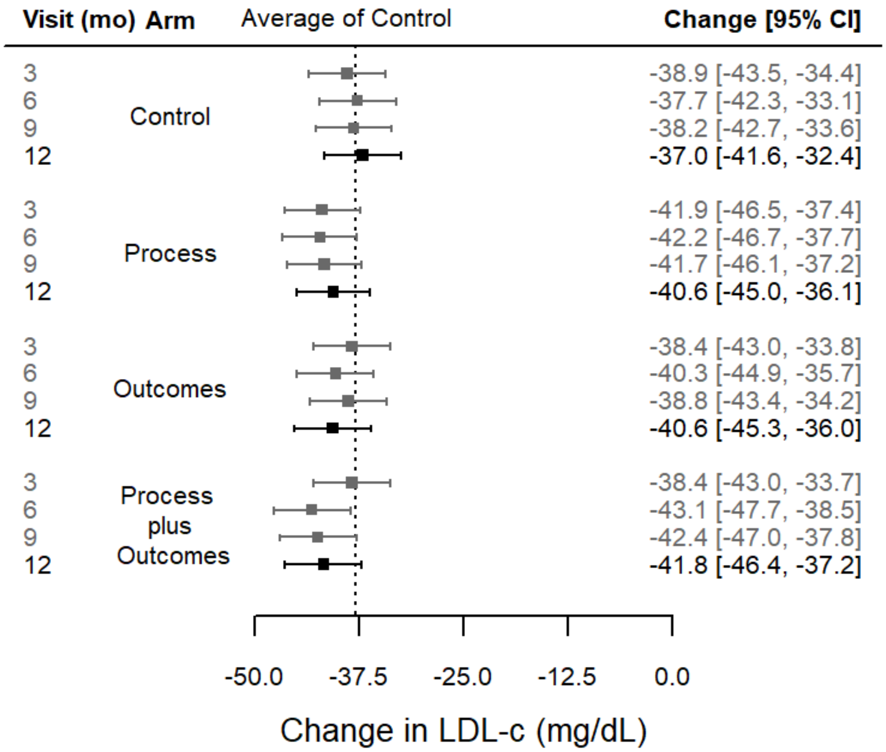

Figure: Forest plots of LDL-c for the intervention period. Squares are the mean change in LDL-c at each timepoint. Error bars are 95% confidence intervals (CI) based on the mixed effects model. Bold squares are the primary outcome at 12 months. Dashed line at the overall mean of the LDL-c reduction for control.

The results of the hypothesis test of each intervention arm versus control are Process (p=.166), Outcomes (p=.416), Process plus Outcomes (p=.088). P-values are unadjusted for multiple comparisons.

© 2021 Reese PP et al. *JAMA Network Open.*

eTable 6: Sensitivity of primary results to different models; Change in LDL-c (mg/dL) from baseline to 12 months using a linear model with adjustment for different baseline covariates

Model Adjustment [a]	Metric	Control arm	Process arm	Outcomes arm	Process plus Outcomes arm
None [b]	Δ_{LDL}	-35.2 (-41.9, -28.6)	-42.0 48.3, -35.7)	-40.0 (-46.4, -33.6)	-43.6 (-50.0, -37.3)
	Difference versus control		-6.8 (-16.0, 2.4)	-4.8 (-14.0, 4.4)	-8.4 (-17.5, 0.7)
	P-value		0.149	0.310	0.069
Baseline LDL-c only [c]	Δ_{LDL}	-36.8 (-41.8, -31.9)	-39.9 (-44.4, -35.4)	-41.5 (-46.0, -37.0)	-42.7 (-47.2, -38.1)
	Difference versus control		-3.1 (-9.9, 3.7)	-4.7 (-11.4, 2.0)	-5.8 (-12.4, 0.7)
	P-value		0.370	0.168	0.081
Site only [d]	Δ_{LDL}	-35.2 (-41.9, -28.6)	-42.1 (-48.4, -35.8)	-39.9 (-46.3, -33.5)	-43.6 (-50.0, -37.3)
	Difference versus control		-6.9 (-16.1, 2.3)	-4.7 (-13.9, 4.5)	-8.4 (-17.4, 0.7)
	P-value		0.144	0.318	0.069
Baseline LDL-c, sex, race, income, education [e]	Δ_{LDL}	-37.1 (-42.0, -32.2)	-39.8 (-44.3, -35.2)	-41.5 (-46.0, -37.0)	-42.6 (-47.1, -38.0)
	Difference versus control		-2.6 (-9.4, 4.1)	-4.4 (-11.1, 2.3)	-5.5 (-12.0, 1.1)
	P-value		0.441	0.197	0.102

[a] Analyses used imputed data

[b] Linear model including terms for intervention arm (no adjustment for site or baseline LDL-c). Mean (95% CI) change from baseline to 12 months. Values for change from baseline LDL-c (Δ_{LDL}) are across all subjects.

[c] Linear model including terms for intervention arm and baseline LDL-c only. Values for change from baseline LDL-c (Δ_{LDL}) are across baseline LDL-c

[d] Linear model including terms for intervention arm and site (Penn Medicine versus LGH) but no adjustment for baseline LDL-c. Values for change from baseline LDL-c (Δ_{LDL}) are across site.

[e] Linear model including terms for intervention arm, baseline LDL-c, sex, race, income, education. Values for change from baseline LDL-c (Δ_{LDL}) are across all terms in the model.

© 2021 Reese PP et al. *JAMA Network Open.*

eTable 7: Change in LDL-c (mg/dL) from 3-month visit to 12-month visit

Timeframe	Group	Mean (95% CI)	Difference from control, mean (95% CI)
**3-6 months**	Control	2.9 (-2.5, 8.3)	-
	Process	-0.3 (-5.9, 5.3)	-3.2 (-11.0, 4.6)
	Outcomes	-2.3 (-7.5, 2.9)	-5.2 (-12.3, 1.9)
	Process & Outcomes	-5.6 (-11.3, 0.1)	-8.5 (-16.3, -0.7)
**3-9 months**	Control	1.8 (-3.5, 7.2)	-
	Process	0.0 (-5.5, 5.5)	-1.8 (-9.4, 5.8)
	Outcomes	0.7 (-4.9, 6.4)	-1.1 (-8.5, 6.3)
	Process & Outcomes	-4.1 (-9.6, 1.3)	-6.0 (-13.7, 1.8)
**3-12 months**	Control	3.8 (-2.0, 9.5)	-
	Process	1.8 (-3.6, 7.2)	-2.0 (-10.0, 6.0)
	Outcomes	-2.2 (-7.5, 3.1)	-6.0 (-13.9, 1.9)
	Process & Outcomes	-4.9 (-10.2, 0.4)	-8.7 (-16.2, -1.2)
*All analyses were adjusted for LDL-c at baseline and stratified by site, using multiply imputed data. Results are presented for Penn Medicine site participants			

© 2021 Reese PP et al. *JAMA Network Open.*

eFigure 4: Secondary outcome of mean weekly measured statin adherence, by intervention arm[a]

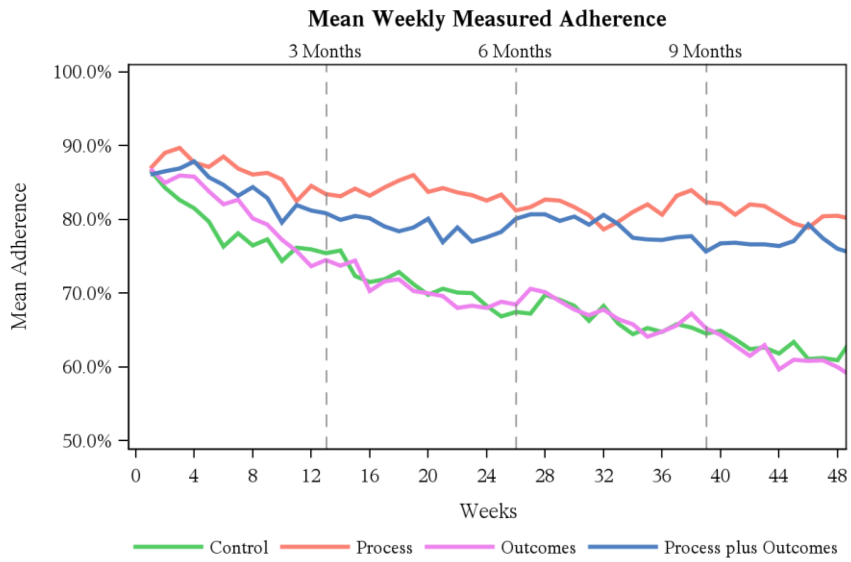

[a] Adherence measured through smart pill bottle. Values are weekly mean adherence for each intervention arm over the 12-month intervention period. The Process and the Process plus Outcomes arms received financial incentives to encourage statin adherence.

© 2021 Reese PP et al. *JAMA Network Open.*

eTable 8: Secondary outcome of mean weekly measured statin adherence, by intervention arm[a]

Window	Metric	Control arm	Process arm	Outcomes arm	Process plus Outcomes arm
0-12 months	Adherence	0.70 (0.66, 0.74)	0.83 (0.79, 0.87)	0.70 (0.66, 0.74)	0.80 (0.76, 0.84)
	Difference vs. control		0.13 (0.08, 0.19)	0.00 (-0.05, 0.06)	0.10 (0.04, 0.15)
	P-value		<.001	0.924	<.001
Final 30 days	Adherence	0.62 (0.57, 0.67)	0.80 (0.75, 0.85)	0.60 (0.55, 0.65)	0.77 (0.72, 0.82)
	Difference vs. control		0.18 (0.11, 0.25)	-0.02 (-0.09, 0.05)	0.15 (0.08, 0.22)
	P-value		<.001	0.601	<.001

[a] Adherence measured through smart pill bottle. Mean proportion of days adherent by study arm across 12 month or in the final 30 days of the intervention. Hypothesis tests from a linear model with adherence as the outcome compared each arm to control and are unadjusted for multiple comparisons.

© 2021 Reese PP et al. *JAMA Network Open.*

eTable 9: Summary of adverse events*

Data on adverse events were assessed at 3, 6, 9, 12 and 18-month study encounters and could also be reported spontaneously at other time points by participants to investigators.

Arm	Adverse Event	Action	Outcome (at last follow-up)
D	Subject reported serious adverse reactions to statins	Dose modification	Recovered with minor sequelae
D	Chest pain	Hospitalization	Resolved
A	Muscle, weakness, and pain in knees fingers and joints	None	Condition still present and under treatment
A	Muscle pain and weakness in thighs	Dose modification	Condition still present and under treatment
C	Skin rash on legs, neck, chest	Dose modification	Resolved
C	Reaction to statin	Dose modification	Condition still present and under treatment
A	Aches and pains in arms/legs	Dose modification	Resolved
A	Reaction to statin	Dose modification	Recovered with minor sequelae
B	Hives	Dose modification	Resolved
B	Muscle pain and weakness in left thighs	Medical/Surgical intervention	Resolved
D	Cardio myopathy worsened	Dose modification	Resolved
D	Elevated liver function tests	Dose modification	Condition still present and under treatment
B	Muscle pain and cramps bilateral lower extremities	Dose modification	Resolved
C	Radiating pain bilateral upper extremities	Drug permanently discontinued	Condition continues to worsen

© 2021 Reese PP et al. *JAMA Network Open.*

B	Muscle pain and weakness	Drug permanently discontinued	Condition still present and under treatment
C	Muscle pain/cramps bilateral legs	Dose modification	Condition still present and under treatment
A	Chest pain	Hospitalization	Condition still present and under treatment
D	Pain in right thigh	None	Condition still present and under treatment
D	Muscle pain and weakness	Other	Condition still present and under treatment
C	Leg muscle cramps	None	Condition still present and under treatment
C	Joint pain	Drug permanently discontinued	Condition still present and under treatment
C	Muscle pain and weakness	Drug permanently discontinued	Condition still present and under treatment
C	Muscle pain and weakness bilateral arms and shoulder	Drug permanently discontinued	Condition still present and under treatment
B	Muscle pain and cramps bilateral lower extremities	None	Condition still present and under treatment
D	Muscle pain over lumbar back and bilateral knees	Dose modification	Condition still present and under treatment
D	ALT & AST increase	None	Condition still present and under treatment
A	Weakness over bilateral lower and upper extremities	None	Condition still present and under treatment

© 2021 Reese PP et al. *JAMA Network Open.*

D	Muscle pain and weakness over upper left arm	None	Condition still present and under treatment
D	Muscle pain and weakness	Drug permanently discontinued	Condition continues to worsen
A	Muscle pain and weakness	None	Condition still present and under treatment
A	Muscle pain and weakness	None	Condition still present and under treatment
B	Increased liver enzyme activity	Drug permanently discontinued	Recovered with major sequelae
B	Muscle cramping and weakness	Dose modification	Resolved
C	Muscle pain and weakness	None	Resolved
C	Intense Pains	Drug permanently discontinued	Resolved
B	Memory/Concentration issues	Dose modification	Resolved
D	Muscle pain in leg	None	Resolved
D	Muscle/joint pain in lower back and shoulders	None	Resolved

© 2021 Reese PP et al. *JAMA Network Open.*

B	Muscle pain and weakness	Dose modification	Resolved
A	Muscle pain and weakness	Dose modification	Resolved
B	Memory/Concentration issues	Dose modification	Resolved
A	Unable to determine	None	Resolved
B	Headaches	Dose modification	Resolved
B	Unhealthy liver enzyme activity	Other	Resolved
D	Shoulder pain	None	Condition still present and under treatment
A	Memory/Concentration issues	None	Resolved
A	Muscle cramps and light headedness	Dose modification	Resolved
B	Muscle spasms in legs	None	Resolved
D	Muscle pains	Dose modification	Resolved
D	Muscle pain and twitching	None	Resolved

© 2021 Reese PP et al. *JAMA Network Open.*

B	Headaches	Dose modification	Resolved
B	Muscle pain and cramps	Dose modification	Resolved
B	Weakening in legs	Dose modification	Resolved
A	leg and foot cramps	Dose modification	Resolved
C	High glucose	Hospitalization	Resolved
A	Muscle and joint pain	Dose modification	Resolved
B	Muscle pain	Dose modification	Resolved
A	Muscle pain	Dose modification	Resolved
C	Muscle pain	Dose modification	Resolved
B	Dose modification	Dose modification	Resolved
D	Muscle pain and weakness	Dose modification	Resolved
* Adverse events were reviewed and assessed by the Principal Investigators on a monthly basis, and then presented using descriptive statistics to the DSMB and IRB yearly.			

© 2021 Reese PP et al. *JAMA Network Open.*

eFigure 5: Spillover analysis flowchart of Penn Medicine patients with hypertension and diabetes plus usual care data for hemoglobin A1c and systolic blood pressure, respectively, in the pre-randomization period (up to 6 months prior) and later period (9 – 15 months post-randomization)

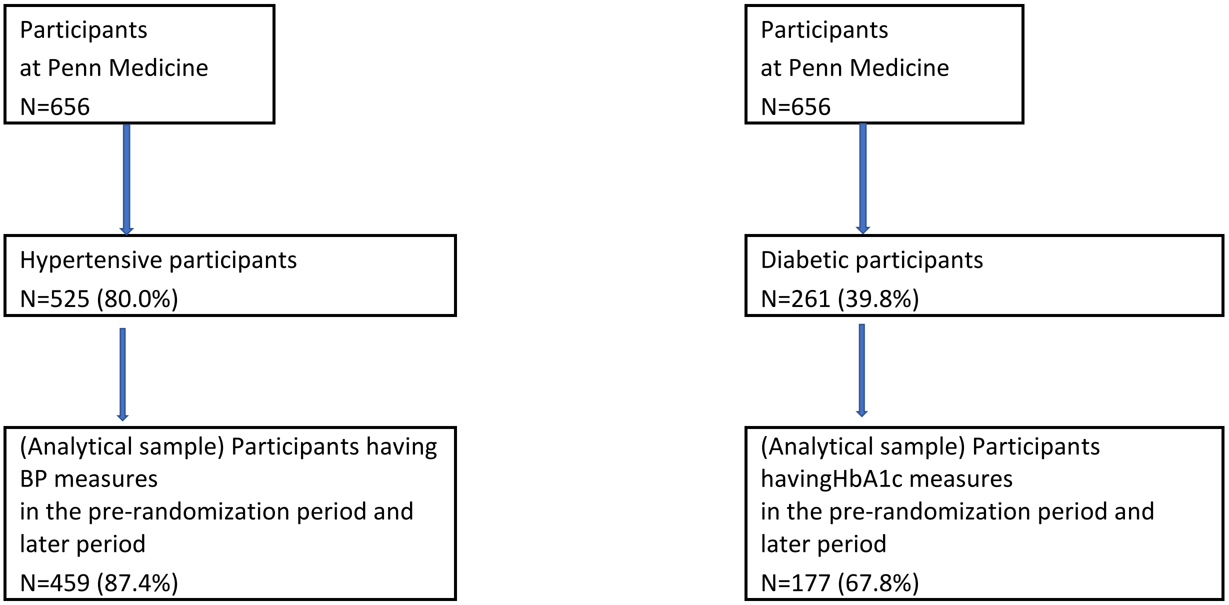

© 2021 Reese PP et al. *JAMA Network Open.*
