# Supplementary material for: Effect of Financial Incentives for Process, Outcomes, or Both on Cholesterol Level Change: A Randomized Clinical Trial
Source: JAMA Netw Open. 2021 Oct 4;4(10):e2121908. doi: 10.1001/jamanetworkopen.2021.21908 (PMC8491106; doi:10.1001/jamanetworkopen.2021.21908)
Supplement: Supplement 3. — Data Sharing Statement [file jamanetwopen-e2121908-s003.pdf]

Data Sharing Statement

Reese. Effect of Financial Incentives for Process, Outcomes, or Both on Cholesterol Level Change. *JAMA Netw Open*. Published October 4, 2021. doi:10.1001/jamanetworkopen.2021.21908

Data
Data available: Yes
Data types: Deidentified participant data, Data dictionary
How to access data: The data will be released to qualified investigators with a request to the PI team at our institution.
When available: With publication

Supporting Documents
Document types: Informed consent form
How to access documents: The form will be released to qualified investigators with a request to the PI team at our institution.
When available: With publication

Additional Information
Who can access the data: The form will be released to qualified investigators with a request to the PI team at our institution.
Types of analyses: For any valid scientific purpose
Mechanisms of data availability: The data will be released to qualified investigators with a request to the PI team at our institution.
Any additional restrictions: None
